# Genome sequence data of *Mangrovimonas* sp. strain CR14 isolated from mangrove forest at Tanjung Piai National Park, Malaysia

**DOI:** 10.1016/j.dib.2020.105658

**Published:** 2020-05-07

**Authors:** Muhammad Ramziuddin Zakaria, Ming Quan Lam, Sye Jinn Chen, Mohamad Hamizan Abdul Karim, Lili Tokiman, Adibah Yahya, Mohd Shahir Shamsir, Chun Shiong Chong

**Affiliations:** a Department of Biosciences, Faculty of Science, Universiti Teknologi Malaysia, 81310 Skudai, Johor, Malaysia; b Johor National Parks Corporation, Kota Iskandar, 79575 Iskandar Puteri, Johor, Malaysia; c Faculty of Applied Sciences and Technology, Universiti Tun Hussein Onn Malaysia, Pagoh Higher Education Hub, 84600 Muar, Johor, Malaysia

**Keywords:** *Mangrovimonas*, Illumina, Genome sequence, Proteolytic activity

## Abstract

*Mangrovimonas* sp. strain CR14 is a halophilic bacterium affiliated with family *Flavobacteriaceae* which was successfully isolated from mangrove soil samples obtained from Tanjung Piai National Park, Johor. The whole genome of strain CR14 was sequenced on an Illumina HiSeq 2500 platform (2 × 150 bp paired end). Herein, we report the genome sequence of *Mangrovimonas* sp. strain CR14 in which its assembled genome consisted 20 contigs with a total size of 3,590,195 bp, 3209 coding sequences, and an average 36.08% G + C content. Genome annotation and gene mining revealed that this bacterium demonstrated proteolytic activity which could be potentially applied in detergent industry. This whole-genome shotgun data of *Mangrovimonas* sp. strain CR14 has been deposited at DDBJ/ENA/GenBank under the accession JAAFZY000000000. The version described in this paper is version JAAFZY010000000.

Specifications table

**Table:** 

Subject	Biology
Specific subject area	Microbiology and genomics
Type of data	• Genome sequence data in FASTA format • Table • Figure
How data were acquired	Whole-genome sequencing using Illumina HiSeq 2500 (2 × 150 bp paired end) platform
Data format	Raw and assembled genome sequences
Parameters for data collection	Genomic DNA was extracted from a pure culture of *Mangrovimonas* sp. strain CR14. The genome of strain CR14 was sequenced by using Illumina HiSeq 2500 platform (2 × 150 bp paired end). The genome was *de novo* assembled using SPAdes version 3.11.1 and annotated using PGAP.
Description of data collection	Whole-genome sequencing, assembly and annotation
Data source location	*Mangrovimonas* sp. strain CR14 was isolated from Tanjung Piai National Park, Johor, Malaysia 1°16′01.7″N 103°30′40.2″E
Data accessibility	This whole-genome shotgun data of *Mangrovimonas* sp. strain CR14 has been deposited at DDBJ/ENA/GenBank under the accession JAAFZY000000000 (https://www.ncbi.nlm.nih.gov/nuccore/JAAFZY000000000). The version described in this paper is version JAAFZY010000000. The sequence data have been registered in the National Center for Biotechnology Information (NCBI) Sequence Read Archive (SRA) database under the accession number SRR11110036 (https://www.ncbi.nlm.nih.gov/sra/SRR11110036).

## Value of the data

• The genome sequence of *Mangrovimonas* sp. strain CR14 provides fundamental knowledge about genes related to proteolytic activity.
• The genomic information of this strain CR14 will be useful for comparative genomic analysis with other *Mangrovimonas* species.
• The proteolytic genes encoded in the genome could be further characterized and potentially benefit to detergent industry for effective proteinaceous stain removal.

## 1 Data description

*Mangrovimonas* is a genus that belongs to the family *Flavobacteriaceae* [1] of order *Flavobacteriales*. To date, only three species were successfully isolated from the marine environment, namely, *Mangrovimonas spongiae* [2], *Mangrovimonas xylaniphaga* [3] and *Mangrovimonas yunxiaonensis* [1]. The genome sequence and algicidal ability of *M. yunxiaonensis* were reported [4]. Besides that, the genome sequences of *M. xylaniphaga* and another two *Mangrovimonas*-like strains (ST2-L15 and TPB-H4) were also studied with elucidation of their xylan and arabinan utilizing abilities [5]. So far, the proteolytic genes of *Mangrovimonas* which potentially to be useful for food processing and detergent application were not revealed.

Strain CR14, a bacterium that affiliated to genus *Mangrovimonas* (99% 16S rRNA gene similarity to *Mangrovimonas* sp. strain ST2-L15), was isolated from mangrove soil samples obtained from Tanjung Piai National Park, Johor. Colony of strain CR14 was orange-pigmented, with 1 - 2 mm in diameter, round shape, smooth surface, entire margin, convex elevation and small size after 48 h of incubation on Marine agar plate. Its genome was sequenced and the proteolytic genes were mined. The genome features of strain CR14 were summarized in Table 1. The assembled genome of *Mangrovimonas* sp. strain CR14 consisted 20 contigs with a total size of 3590195 bp, while the N_75_ value and G+C content was 367,190 bp and 36.08% respectively. Based on the genome annotation, a total of 3209 genes were predicted in which, 3152 of them were responsible for coding specific proteins while 46 and 11 of them were coded for RNA genes (39 tRNAs, 4 ncRNAs and 1 for each 16S-23S-5S rRNA operon) and pseudo genes, respectively.

**Table 1:** General genome statistics of *Mangrovimonas* sp. strain CR14.

Category	Strain CR14	
	Number	Total percentage (%)
Number of contigs	20	–
Genome size (bp)	3590,195	100.00
G +C content	1295,342	36.08%
Total genes predicted	3209	100.00
Protein coding genes	3152	98.22
Non-coding RNA genes	46	1.43
rRNA genes		
5S rRNA	1	0.03
16S rRNA	1	0.03
23S rRNA	1	0.03
tRNA	39	1.22
ncRNA	4	0.12
Pseudogenes	11	0.34

**Fig. 1:**
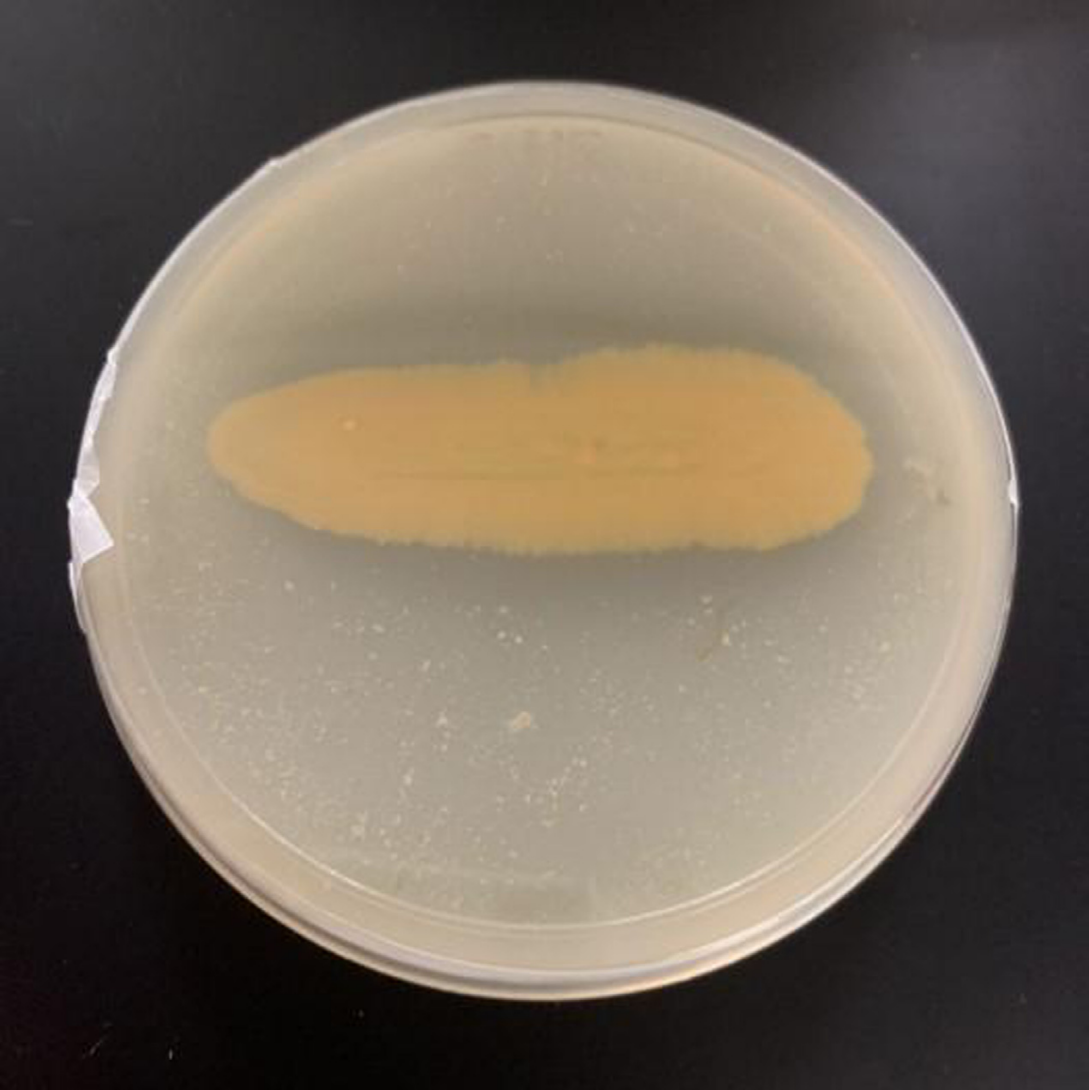
Mangrovimonas sp. strain CR14 positive hydrolysis on skim milk containing agar showing ability of this bacterium to produce extracellular proteolytic enzymes.

Furthermore, a total of 100 genes in the genome of strain CR14 were predicted to be involved in proteolytic activity. In details, 1, 42, 6, 15 and 36 gene(s) were encoded for aspartic, metallo-, zinc metallo-, serine and ATP-dependent proteases respectively. After being subjected to SignalP version 5.0 server [6], 10 proteolytic genes were predicted to be secreted extracellularly. These proteases were matrixin family metalloprotease (locus tag: GZ212_06430), ATP-dependent zinc metalloprotease (locus tag: GZ212_13440), M1 family metalloproteases (locus tag: GZ212_02095, GZ212_11920, GZ212_15430 and GZ212_13780), S8 family serine proteases (locus tag: GZ212_02100 and GZ212_10035), M23 family metalloprotease (locus tag: GZ212_14610) and P1 family proteases (locus tag: GZ212_15520). In addition, *Mangrovimonas* sp. strain CR14 was also tested positive on skim milk containing agar (clear zone was shown) further proved the ability of this bacterium to produce extracellular proteolytic enzymes (Fig. 1).

## 2 Experimental design, materials, and methods

The inoculum of *Mangrovimonas* sp. strain CR14 was prepared. A 0.5% (v/v) of strain CR14 glycerol stock was aseptically cultured in Marine broth 2216 at 30 °C for 18 h. Inoculum with optical density (OD_600_) of 0.7 was then streaked onto Marine agar 2216 and cultured at 30 °C for 48 h. Genomic DNA of strain CR14 was extracted and purified by using the Quick-DNA Miniprep Plus kit (Zymo Research) and DNA Clean and Concentrator™-25 (Zymo Research) respectively according to manufacturer's instructions. The quantity and quality of the purified genomic DNA of strain CR14 were then checked using both Nanodrop™ spectrophotometer and Qubit® fluorometer respectively. The library was constructed by using Nextera sample preparation kit [7]. The whole genome of strain CR14 was sequenced on an Illumina HiSeq 2500 platform (2 × 150 bp paired end) with a genome coverage of 155 × . Sequence adaptors and reads with low quality scores were filtered using BBDuk of the BBTools Packages [8]. The filtered reads were *de novo* assembled using SPAdes version 3.11.1 [9]. The genome of strain CR14 was then annotated using National Center for Biotechnology Information (NCBI) Prokaryotic Genome Annotation Pipeline (PGAP) version 4.11 [10].

## Appendix Supplementary materials

Image, application 1

Image, application 2
