# Supplementary material for: Genome sequence data of Mangrovimonas sp. strain CR14 isolated from mangrove forest at Tanjung Piai National Park, Malaysia
Source: Data Brief. 2020 May 7;30:105658. doi: 10.1016/j.dib.2020.105658 (PMC7225383; doi:10.1016/j.dib.2020.105658)
Supplement: Supplementary file 1 [file mmc1.docx]

***Mangrovimonas* sp. CR14 List of Genes Encode for Proteases (FASTA)**

Extracellular proteases that could be secreted extracellularly were highlighted in yellow

>1. Clan AA aspartic protease

MENNLRDYLVDKGYTKIKLHLTKTNHFEIRASINGVKGVFILDTGASNSCVGFEAAETFKLKTMESEIKAAGAGAIDMETQLSKKNKIKIGKWKKDKVALILFNLTHVNTALVNHNAQPVDGILGADILKKGKAIIDYDKKYLYLKL

>2. RIP metalloprotease RseP

MEFVIKISQFLLSLSLLIILHEMGHFIPAKAFKTRVEKFYLFFDIKFSLFKKKIGETVYGIGWLPLGGYVKISGMIDESMDTEQMSQEPQPWEFRSKPAWQRLIIMLGGVTVNFLLAIVIYIGMSYYYGDKFLPNDNISDGYVIESTVANKAGLKTGDKIIAVDGEKIENFAEVPEKVLFGKDITILRNGEQSTITLPQDFLAQLIDAKEKGFIDLRMPFIIAQVPDTSANAQSGIRKGDVIVGFNDTDTKYIDQVVSSLDSYKGQQVTAHILRDHEKLNVPLKISDDGKLGLVYATRATPETLEELGYYEYDVKKYGFLESIPVGFSKTKERLVSYVKQVKAIFTPSTGAYKGVGGFKAIYDIFPTVWSWQYFWGITAFLSIMLGFLNLLPIPALDGGHVMFLLYEMVSGRKPGDKFLEYAQMVGFVILIALVLFANGNDLFKAIFD

>3. CPBP family intramembrane metalloprotease

MTLKNKKHSWLLISGIGMFCLGMVTSFFIDFYETEIESYTKASFPVFLFIAIILAPIFEELSFRGGFVQSKILRATSLIGLVALFFITQSYLTKVITLIYLVLLIVSFFKKEVSLLVPLALLNAFLFALFHLDTDHLISWNALAGFSFRSAFAFFAIWICINFNLLKSILFHSVWNLSLMLVFSYSLFFPDETIQHYENDNIQVTWNRTSKKFHGEVNFPTMNHIKATNCEAPFLLKSVLWSDSTASKTNHLLPSEPFMGYDFEIKLKDTLSKRPNLYQPIKQFLLDEELLLITNPEEH

>4. Zinc metalloprotease

MKKVILSMAALAMIFATSCSNDSKETMNTDQQAKVDMSDFYLYTDADIDEQSRVASGQKSCHSMVNLNRLLNENPGLEQTMYDIEYNLRAAIAAKKPDGAGNGNGNGGGNNGGGTDPDPGFTDPVSIPVVVNIIEQYSNQVTTQQINSQIAVLNEDFNNNNSNTSGVPSEFSGAVADCGISFTLHTVNRVVSTQSSWGTNDAMKHSSQGGIDVTDPSTYLNIWVCEIGGGILGYAQFPGGPSSTDGVVIGTDFFGENAAGGVYGHGRTATHEVGHWLNLRHIWGDGQCKQDDFVQDTPPSNGPNYGCPSYPTVNCQTADMTMNYMDYVYDDCMYMFTNGQNTRMRALFESGGARASLAGN

>5. Rhomboid family intramembrane serine protease

MKEDSFQFSNGVVVYPLLFVLSIWVVFWAEVRFGFNFTRLGIYPLAVKGIPGIALSPLIHSGLTHLLHNSLPLLILSAALFYFYRPIAWKVIIFGILLSGLMTWLIGRPSYHIGASGLIYVLVSFTFFKGIFAKHYRLIALSLMVVFLYGGMIWYTFPIKDEISWEGHLSGFLVGLFFALVFRKQIAKPKKYEWEKDDFNEDDDPFLKHFDADGNFIEQLPEEVDLEEDTQDSIVYNYIYKKKDDDD

>6. Tail-specific protease

MKRNYKVLMLVLLLAFASCSFTTKTFDDPDKDKLLVQVITYVLQRGHFSPRDFNDDFSEEVYNNYLDQIDPLKRYFYKSDIEEFEKYKYELDDQLKAYDISFFSLTHERLIKRIEEAKAIYKDVLDKPFDYTVNETYSSDYENESYVSSKKEMRDRWRKQLKFTNLANYDDLIVMQENDSTSTDKKSMSELESKSREETLKSINMYYTDYVDDLARKDWFAMYVNAIVSEFDPHTFYFAPEDKERFDQQMSGKLEGIGARLQKRMDNTKIVELISGGPAWKGKELEVGDIIMKVRQEDEDQAVNIVGMRLDDAIKLIKGPKGTKVTLTIKKVDGTIKDINITRDIVELEETYAKSAMVGKDDKKFGVINLPKFYVDFEDYKNRNAASDLKKEIERLKSEGMEGLVLDLRNNGGGSLQTVVDIAGLFIKDGPVVQVRSNEEKEVLKDDDRSIVWDGPLVILVNELSASASEILAAAMQDYKRAIIIGSKQTFGKGTVQNLYDLNRMVRNNQEGDLGALKITTQKFYRINGGSTQLEGVKSDVVVPDRYSFINIGEKDQQNPLPWDKIDAVDYDLWDSYFDYDTTISNSKKRMADNEQLKLIEENAKWVKARMDETQHSLNYEAYKAMLEKNEEEAKKFDKISKYETNLVFESLPYEQKLFANDTVLKDRRDSWHKSLSQDVYIEEALNVLEDLKMTYSIKKVAASSVKE

>7. CPBP family intramembrane metalloprotease

MKKIALVPAFLIFLVWFFFTFGLQFVMGTHESGIDLSEIVSKQLNYGIFLSALTCLIFAYFGKHQIRVGLSGSRKFTDKVIIIPIIFIIIAQIGAFTGGAYSNSSFVTWVLINTLFVGISEEMMFRGIFMSSFTNKFGYWPAVIWTSLIFGGIHVLNGFITGDFAMATLQAFMATCSGLLFLAIRVRTLSIITAIILHWIWDFTVFISGTVITPTEDLVSLIPSMLLVIAPVLFGIIGILSLRNKKAAEAYVASQAEG

>8. ATP-dependent Clp protease proteolytic subunit

MSKTTKVQDLLDHKLLEERKVFLWGQVDDDSAKHVIDRLLYLDALETKDIHLYINSPGGYVTSGFAMYDCIQSLKSDVSTICTGLAASMGSILLSVGAKGKRFIQPHAKVMIHQPSGGARGPASDIEITAQEILKTKELSAKILADNCGQDYEKVLKDFNRDHWMDAEESVAYGIVDAVI

>9. ATP-dependent Clp protease ATP-binding subunit

MDDNFSPRVKDVIAFSKEEALRLGHDFIGTEHLMLGLLRDGSGKAINILNALDVDLSHLRRKVEILSPANPNVAIASNEKKNLHLTRQAERALKTTFLEAKLFQSSSINTAHLLLCILRNENDPTTKLLNKLKVDYDNVKEQFKFMITNDEEYIEPRAEAFDDDAPEDDTSKGDIFNTPSSKSNKKSKTPVLDNFGRDLTALAEEGKLDPVVGREKEIERVSQILSRRKKNNPLLIGEPGVGKSAIAEGLANRIIKRKVSRILFNKRVVTLDLASLVAGTKYRGQFEERMKAVMNELEKNDDIILFIDEIHTIVGAGGATGSLDASNMFKPALARGEIQCVGATTLDEYRQYIEKDGALERRFQKVIVEPTSIDETIEILNNIKEKYEDHHNVIYTPEAIEACVKLTSRYMTDRFLPDKAIDALDEAGSRVHIKNIDVPKQILDLEKQLEEVKESKTSVVKKQKYEEAAKLRDDEKRLEKELAVAQEKWEEETKLHRETVTEDNVADVVSMMTGIPVNRIAQTESNKLAQLPEMIKGKVIGQDEAVAKVVKAIQRNRAGLKDPNKPIGSFIFLGQTGVGKTQLAKVLSRLLFDSEDSLIRIDMSEYMEKFAISRLVGAPPGYVGYEEGGQLTEKVRRKPYSVVLLDEIEKAHPDVFNMLLQVLDDGYLTDSLGRKIDFRNTIIIMTSNIGSRKLKDFGTGVGFGTASQKAQEDANARSVIENALKKAFAPEFLNRIDDVVVFNVLEKEDIDKIINIELEKLFVRIKNIGYDLELTDSAKGFIADKGFDKQYGARPLKRAIQKYVEDTLAEEIINSKIEEGDKIILDLDKKTQDLTVTIKKTKENTTES

>10. Matrixin family metalloprotease

MRLASWLLLILLFSCSKRQSDSVIIGVQAYGEVNPAFLDSISDALETSFANEVVVLNQLSLPDHAFVHIKNPRYRADSLLRDLVRNKPWYVDHVIGVTNHDISTTKKNDLGEIKKPVYKYMDWGVYGLGYVDGPSCVVSTYRLGKTCEPQFYARLKKIAIHEIGHNLGLNHCENKLCVMQDAAETIATIDRVGFNICSDCKRIIK

>11. CPBP family intramembrane metalloprotease

MYITQAFNVLHDWWRYLIGLLVVIFAVIIGQIPFTVAVFFKAFESGENIFGMDETQMMGMLESNLNLFLMLLSFAAGLVGVFFVVRTIHKQTITSLTTARKKIDWGRILFAFLLWGILSSSFVVADYYMNPENYQWNFKMIPFLILLLISVVFIPLQTSFEEYLFRGYLMQGIGVISKTRWLPLAITSLIFGLLHIANPEVEKMGYIIMIYYIGTGLFLGIMTLMDDGMELALGFHAANNLFTALLVTADWTAFQTHSILKDISNPTAAGFFDVFLPVFVLFPILLFIFSKKYRWTNWKDKLFGHVEEPIKEDYKILD

>12. CPBP family intramembrane metalloprotease

MKEKTFRLIEFFILFVIFPVSFAMDYPVAIKVSLGVLGFLYVLWVMIKLEGVTFFIQKEIHWSVFIKRTLVKFFVLAVFTIIFVLLTDREHLFTFMMEKPLYWFLFTLVYILFSVYPQELLYRTFFFKRYSDFFKSEALLIFMNAILFSLAHLFFRNTMVHVLTFIGGVIFALTYQRTKSTLLVSIEHALYGSWLFMVGMGGMLGFPT

>13. ATP-dependent Clp protease adaptor ClpS

MSIKEKVSVDVLLEEEVQNQNEIVLYNDDVNTFDHVINTLIYACDHTPEQAEQCSIIVHYKGKCTVKTGDFDDLRPRCSMLLDAGLSAEIV

>14. Rhomboid family intramembrane serine protease

MNLRISETVKHLLIINVVMFIGTIIVGDGMLFNRWFSMFFPKNNWFEPWQIITHMFMHGGVTHILFNMFALWMFGTPVEQRLGSKKFLFVYFSAGLGAVILQVGYSYFQFNNLISPLVEHGYTMKEIVAALNSGGLNKSVVNPIIGPDNLDSLNNIFYGRLVGASGCIMGILAAFGVLYPEAKLMMIFLPIPIKAKYFIPGIILLDLISALSGNSFFSPSNTAYMAHVGGALVGFFIMWYWRKNQFNNNRWDL

>15. Rhomboid family intramembrane serine protease

MTSLNQDISSKLKNLNGLEKLIAVNVFVFFIVTILRALVGRFMILDWLELPSDFYSFLIKPWTLVTYSFLHYDFFHILFNMLWLYFVGKMFLTLFNVKLGMNVYFLGAISGGLLYLLGYNVIPSFFSGPSRLVGASAAVRALLIFICAYSPFMDVRMFTFKIKLWHIGVAVVAIDILGLFGANSGGNLAHLGGAILGYGYAVQLRKGNDIGKGFEKMMDSLSSLFSKKEKSPLRTVHKTRGRKEKMAGYSKEEFSEFNTQKKIDVILDKISKSGYDSLTKEEKDFLFRAGK

>16. CPBP family intramembrane metalloprotease

MIGILVAIAISWLLLYFIENKTLSVLGILPTPKRLKQFFLGFLITGILCVAVQYLEAHLKSSTWILNDNITSAIILKSLWWDFKSVFTEELIFRGALLYILIQKIGPSKGILISAIAFGVYHWFSYGVLGNVMAMILVFIGTGIMGYAWAWAFSKTKSMMLPFGLHLGWNVIYNTLFSKGPLGELILVSKSGNELTDWASLLNFISGLVLVPILIIIYIKYFVKKEP

>17. ATP-dependent zinc metalloprotease FtsH

MAANKNNPKNKKPKFSPYWIYGILIAMFIGLQLFSGGGLQDASKTTPSQFIQFLRDGDVSKVVIVNEKYARVYLTPEAEKKEVHKKSINKSLLPSATKAPNYTFRFGAIEIFQKQLEDVSSEISPTPTIEYKEEQNYWGDFLISMLPFILLIGVWIFIMRRMSAGSGGGAGGQIFNIGKSKAKLFDENTEVKVSFKDVAGLEGAKEEVQEIVDFLKNPEKYTSLGGKIPKGALLVGSPGTGKTLLAKAVAGEAQVPFFSLSGSDFVEMFVGVGASRVRDLFKQAKEKSPSIIFIDEIDAIGRARGKNNFSGSNDERENTLNQLLTEMDGFGTNTNVIVLAATNRADILDKALMRAGRFDRQIFVDLPDVRERKEIFEVHLRPLKKDKDLDLDFLSKQTPGFSGADIANVCNEAALIAARKGKKSVGKQDFLDAVDRIIGGLEKKNKIITPEEKKAIAFHEAGHATVSWMLEHAAPLVKVTIVPRGRSLGAAWYLPEERLIVRPEQMLDEMCAAMGGRAAEKVIFDKISTGALSDLEKVTKQARAMVTIYGLSDKVGNLTYYDSSGQNDYGFTKPYSEKTAELIDKEISDIVEEQYQRAINLLTQHKDKLTELAEVLLEKEVIFKDNLEKIFGKRPFAKEEELAEMDNLEDNNEPIEVNEEK

>18. PrsW family intramembrane metalloprotease

MLNLILAALAPVVAIIVYIYAKDKHEKEPRRLMFYTFLFGAFLSIFITTVLYVIFDYILPINKDTVLQQFIKAFFIVGLTEEFSKYIIVRYYSQRRLEFNEPFDGIVYAVMVSMGFAATENILYVLEGGYEVALLRAFTAIPAHATFAVIMGFFMGRAKFSKNRIKWNLIGLLGAIIFHGAYDFFLFIHFIPGIWIGAFASLILGLFLSLKAIKIHQEGSFFKNT

>19. Rhomboid family intramembrane serine protease

MGNLSIVTIIIIAANVLISYKGFGDFGFFEKYKFNVGAIRRGEQIRMFSSGFLHVDVAHLVFNMFTLYIFANVVLAYLGNINFIIVYVGSLLLGNLLSLYFHKNEYYYSAVGASGAVMGVIYSAILLRPDLTIYLFLVVPIPAYIFGIAYLLYTIYGMKSRVGNIGHDAHFGGAIGGYVITLMLASWLFETHLTMIILLLIPIVLLFVLKKMNKI

>20. ATP-dependent Clp protease ATP-binding subunit

MAKEELECSFCGRKKPETNLLIAGLDAHICDRCIEQAHGIVLEESKQSHNTELSAELMLRKPQKIKQFLDEYIIGQEATKKVMSVAVYNHYKRLLQSPTDDDIEIQKSNIVMVGQTGTGKTLMAKTIARMLNVPLAIVDATVLTEAGYVGEDVESILTRLLQAADYNLEKAEKGIVFIDEIDKIARKSDNPSITRDVSGEGVQQALLKLLEGTVVNVPPKGGRKHPDQKFIEVNTENILFIAGGAFDGIEKIISKRLNMKAVGYSASMSDDQIDNDNLLQYIIPKDLKDFGLIPEIIGRLPVLTYMNPLDDKTLRAILTEPKNAIIKQYKKLFEMDDIDFSITDGALDFIVEKAVEYKLGARGLRSLCEEILTDAMFEMPGTNDKELKVTKAYAEDKLTKTTLKKLKAVS

>21. Membrane protease subunit, stomatin/prohibitin

MLGINYIKFDSMNYVIHYEAGKIKKEGRGLSFFYFSPNSSIVSIPVQSDDFQFVFNETTKDYQEVTLQGQVTYKILNPKQLAENLDFTVNNKKQYLKNDYEKIQQRIINEAQTASASVIQRLSLKEALRKLDEIEAEIFTSIQKSKTVQMLGLEILSVNVLAVTPNPEMARALEAQMRESLQKEADQAIYERRNFAVEQERMIRESELNTEIAVEEKQKQIVEKKMETDVVKQQNEQKLKEMEMSSNISLEEKKKGLIDIQVANEKKEADMKEYVLNANLKPYKEMDWKTLMAISNSGNNPTNNIALAFRELAENADKIGNLNISPELLDSILANK

***Mangrovimonas* sp. CR14 List of Genes Encode for Peptidase**

>1. Line 2742: /product="S41 family peptidase"

MKFNKTYIPLFLGIAIAFGIYIGGNLDYADANDRLFSSNSKKDKLNRLIDYIDYEYVDDVNTDSIVDVTVNGILENLDPHSVYIPKEEMERVTDNMKGDFVGIGINFYTFRDTITVIRSVEGGPSDRAGIKGGDRIIMADGDTLYGKDIENDDIIRKLKGPVNSTVRLTVYRKDEDRLLNFKIKRDHIPIASVDAAYKLTESLGYIKINRFAESTYKEFKKQLTKLQDQGITELVLDLRENPGGFLGVAEQIVDEFLEDDKLILFTKNKRGDIEKSYATKKGDFEDGEVYVLIDENSASASEIVAGALQDNDRGTIVGRRSYGKGLVQREMDLGDGSAVRLTVSRYYTPTGRSIQRSYTNGNQDYYDEYYERLERGELLDGSKIEVHDSLKFTTPKGKVVYGGGGIIPDVFVPLDMGMQNETLTYLQRRGFISNFVFEYLEADRHAYDAYDRDTFIDSFVTDDDLVLEFQNYLNDKTRANITFIAYNDEVKQYIKANLADQLYGKGAYEEILNQNDIMIDEVIQLSQDKP

>2. Line 3401: /product="carboxypeptidase-like regulatory

MRINKLFLLFFFLHLVGFSQDIKGVVFDSETGEPLEGASVYFDNTTIGTTTNEKGEFSLNIDESVRSALIISFIGYEKVVMEEYKPGSILKIQLTENVASLNEVNLSFNDGWTRKQKLQAFKRYFLGNSENALSCEIKNEDALILRYLGKSNQLVASSKEPLIIENKRLKYRISYDLQDFNIQYEKNDTFGQVFPISVFYAGTSYYQTDENWDGSKKIINRRKEVYFGSSLHFMRSLVKGKLHEEGFRIFYKKTEIAQTGVFAVSDTDKTNIKEIKIFKQPLLIVYNNNFNNQSSITTKEDIFYVDEFGNHMPADAVMFTGYFANQRIGDSLPLNFEADK

>3. Line 3601: /product="LD-carboxypeptidase"

MSKKALILSVFCVVFSFGIPKVKAQSTTNLKTQTLIKPPYLKVGDTVAIVAPSGILKNREAEINRAKKLLQSWGLHAVVGKHVFSQANHFAGTDDERCEDLQKVLDDPSISAIWCARGGYGTVRILDKLNFSKFKAHPKWLIGYSDITALHNAIHNLGVESIHAMMCTSLTGDKTDLDQTIETFKKALFGESLSYHLDASQYNQEGVVMAPLVGGNLTMLHTMLGSTTSIDTSGKILFIEEIGEYKYHIDRMLQSLKRAGYFDHCVGVVVGDMTKLKKNTTVWGTSIEQLILDALSDYDFPIAFNMPSGHEDDNRALILGRTVELTVSKNGSTLNFKSE

>4. Line 3881: /product="M28 family peptidase"

MLRKIISVLLIALAIYWSFKALLPNTISTKEAASNQFSTARALEKLKPMSLAPHFLGNKEHESVRNYLVNELRALGLKPEIQEGYSMSQWGNLAKAKNILARIKGSQNTKALLLLSHYDSNPHSSLGASDAGSGIVTILEGIRAYMADGKKPKNDIIILFSDGEELGLNGADIFVNQHPWAKDVGLVLNFEARGSGGPSYMLVETNQGNSLLMKGFVEAHPEYPVANSLAYSIYKMLPNDTDLTRFREDGNIDGFNFAFIGDHFDYHTALDNFERLDRNTLEHQGSYLMPLLSYFSQADLTQLKSNDDHIYFNVPFLKTVTYPFSWIFPMLIIAFGLFLWLLIYGFRNQKLSGKWLGKGFLAFFGSLILSGGIGFFLWKLLLTIYPQYGEILHGFTYNGYTYIAAFISLAMMISFFIYSKFHTETKLASLFVAPLTFWLLICTIAAFALEGASFFIIPVYFGLLSLFIMHRQQKPNLILLTLLTVPVLMIMSPFVKMFPVGLGLKILFVSCILTVMIFGLVVPVLGSIKHKNRFTIVFGVLTLWFLFSAHFNSDFSEDRPKPNSLVYLLDSDTHTAQWATYDHLLDDWTQNYISQEKMEVQSENQNVFASKYSTGLTYTHKAPVKMLPEPTIEYIQDTIINGFRNIELILISNRSAERIEVFSDSTSVFKNTKVNGVLAFSDSLGYNFSERKNNRLFSYYVSDEEPLKLSFSVPENQATTLQFYEATFDLLTNKKITIPQRKSNMIPKPFVLNDAVIIKKTVQIN

>5. Line 3984: /product="M13 family metallopeptidase"

MNRKIMNASVLAAGVLVGLSSCKEEKKEMAEVYVAPGIELKNMDTLVKPSEDFFKYVNGTWLENNEIPADRTRWGSFDELRQMTDEDALGILKSAMASDNKDMAQIKVMPGSDQEKAIKIYETIMDTVARDNQGLEPIKPYLAEIDGISNLADLQAYLIKMEPQGGAGFLGFGVGSHPKNSDINAAYLGNGSLGLSRDYYVDQDEDTKQKRDLYKAHIATMMKYLGVDDATATQKAENILAFETRLAEPRMTKEERRDARKRYNPKSLEGLKEMTPSIDWNTYFQGIGVQSVDTVIVSDPGYFKALDGILKEGNVQDWKDYLTWTLLNGASGKLTTELDRVSWEFYGRDLKGSKEQRERDERALQTINWTVGEALGKLYVEQKFPAEAKAKAEAMIKNVMLAYGNRIKNLEWMSPETKEKAIEKLEKMTVKIGYPDKWKDYSQMEVKTYEEGGSYYDNMTNVSKWRFNKDLEDLGKPVDKSEWGMSPQTVNAYFNPSYNEIVFPAAILQPPFYNYEADEAVNYGGIGAVIGHEISHCFDDSGSRYDANGNLNNWWTEEDLEQFTKLGKELAGQFSNEEVFPGVNLNGEFTLGENIGDLGGVNAAYDGLQMYLAENGNPGEIDGFTPEQRFFLSWATVWRTKYRDDALRNQIKTDPHSPGMFRAVMPLKNVDAFYSAFDVKEGDAMYLAPEDRVRIW

>6. Line 5945: /product="U32 family peptidase"

MQQIELMAPAGNFESLQAALDNGANSVYFGVEQLNMRARASINFTLDDLPEISRRCQEKGVRTYLTLNTIIYDHDLSIVKTLVKKAKEANITAVIAMDQAVIAMAREHQMEVHISTQINITNIETVKFYTMFADTMVLSRELSLRQVKKITEQIEKEQIKGPSGRLVEIEIFGHGALCMAVSGKCYLSLHSHNSSANRGACKQNCRKKYTVIDQESGFEMEIDNEYIMSPKDLCTIDFLEEVVDAGIKVLKIEGRGRAPEYVAQVIKCYREAIDSIEEGTYSKEKVIGWMQDLEKVYNRGFWSGYYLGQKLGEWSKGSGSHATQKKVYIGKGVHYYPKTSIAEFKIEAYDLSLGDTILITGPTTGSKEITVDTMLVNDEQLEKGGKGDSVTIPIPFRVRPSDKLYKIVENKVEA

>7. Line 6254: /product="peptidoglycan DD-metalloendopeptidase family

MRHNQLATYFVIFLMCFTSSVFSQSKKQKELEERRQELQREIRQINDLLFKNQSQKKSQTSLIEDLSYKVNVRKNLIKVTNQQANLLTREINANQKKISELRDELTTLKENYAKMILRSYKSKSEQSRIMFLLSSSNFKQAYKRIQYMTQYSKFQKEQGEAIKVKTAELQKSNKELLRQKDDKQKLIAENRVAQRELEGELRQQQSLMASINQNLSKYRNQIKEKQKEADRIDKEIEKMIREAIASSNKKAGKSTNTSSFALTPEDKALASSFVANKGKLPWPVEKGVVKVRYGKQPSPIDRTITIQSNGVRIATEKDADVRAVYKGKVLRVQAIRHGNVLVMIQHGNYITNYTNLSKVFVNPGDMVDTKQVIGKVFTNPSNGETTLKFSIWKEINTQNPADWIFQM

>8.Line 7266: /product="M23 family metallopeptidase"

MTAEKKKSKKIKKKLLHKYRLVILNEDTFEERLSFRLNRLNVFVLMSLTSIFLVLGTIFLIAFTPLREYIPGYSSTALKEKAVELNYKTDSLQRLMAINEKYFESIRKVLHGEVSPQEFNRDSTIEDAKLDASVINLKPTKEDSLLRLKVEKEDKYNLFETATSTINFVLFPPISGSISQEYNFDEKHYAIDVIAPENTPIKATADGTVIFAEWTAETGYVMIIEHGNGLISVYKHNSSLNKTQGELVKSGEVIATIGDTGELSSGPHLHFELWKDGYPINPTNFIDFN

>9. Line 8016: /product="S41 family peptidase"

MKRLLKTRIIIPILALAILVTASSFKNDFFEVAKQLEIFTTLFKEINMSYVDETNPADLMDKAIKSMLEDLDPYTQFYNEQDVEAEKIRRTGDYTGIGAKVRVLKDKLLIVEPYQGYPADKAGLKAGDEIIKVNNTVVADFKDDAGELLKGAANSSANVTYVRQGKQYTTEIKHSEIEIDAVPYFSMINDKTGYIVLNAFNTKASAQTSYALRDLKAQGAKKIILDLRGNPGGLLHEAVNIVNLFVPKGQLVVTTKSKVEKFNRTYYTSKDPVDTEIPLVVLINGSSASASEIVSGSLQDLDRAVIVGSRSFGKGLVQRPKPLTYGTQLKITISRYYTPSGRCIQALDYWHRDEQGNAVRIDKKNYNEFKTKGGRKVYDGGGVQPDEAMEISKNSAVTDAILNDFLIFDYATNYYYKHPNLENIGQFKLGNSDFEDFKKYLKSNDFSFVTETEKALSEVHKKAIKEDLEDNIKNEYNQLLKTLNSSKELAINQNKDQILSLLTDEIVKRYVYREGLYDYYKNHNPEIKKSTEILSNLETYYDYLRLKKG

>10. Line 8098: /product="M1 family metallopeptidase"

MKQTLFLILLSFSLSSFAKIDPPVEQPKTGYWQQHVDYKMNIDMHVDNYQYDGTQTLVYTNNSPDVLNKVFYHLYFNAFQPGSEMDVRSRTIADPDSRVGDRISKLKEDEIGFIKVSSLKQNGTDLTYNVVGTVLEVDLAKPIQPGEKVTFDMVFNAQVPVQIRRSGRNNKDGVALSMTQWYPKMAEYDDEGWHADPYIGREFYGVWGDFDISLTIDKDYVVGGTGYLQNPEEVGHGYQSGKAKKVKGKTLTWHFVAPNVHDFTWAADPEYVHDKLQVENGPMLHFFYKGADSIQENWKKLQPKAADLITFYSNHVGKYPYKQYSVIQGGDGGMEYAMCTLITGGSKLGGLIGTTAHEMAHTWFQFLLATNEVRHEWMDEGFTSYISDVAMNEVMDEGKENPFSNAYKYYAYLATSGREQPLTTHADRYAFNQIYSLAAYVKGEVFLAQLGYVMGEENLNKTIKQYFDDWSFKHPKPMDFIRVAEKVSGLELDWYLVDFGQTTNTIDYGVKEIASKEVTLERIGLMPMPMDLTVTYTDGSTEDIYIPLRIMRGEKPTDATILSDWAWAYPTYTFTAKKEVQSVAIDPKGRMADVNQSNNSKEKE

>11. Line 8121: /product="S8 family serine peptidase"

MRVLKPVLFSAVAAAILSSCGSGIDIVSTPIENVDTTPLKVSELTESEKHNWGHLDLVKDTIPGMSVDKAYSEIIKDNKGQTVIVAVIDSGIDINHEDLDGVVWTNEKEIPGNGIDDDKNGYVDDVHGWNFLGDGYDEQLEYVRILAKGDTSHPDFERAKEEYEKEYQKTVAQKTQYEQIFKQVEAADKAIAAHLGKKDYTPEDVNGITSTDEELNRYVSMMKYMYSNGLDSAEAALEELQGGLDYFSDKLDYNLNKDFNGRKTGDNVDDLTDVGYGNGNVTPSEEGESHGTHVAGIIAAERNNGLGANGVANNVKIMSIRAVPNGDEYDKDIALAIRYAVDNGAKVINGSFGKYYATHSDWVRDAIAYAGEHDVVFVNAAGNEGIDLDKKACYPNDQVDNGPEVSNTFITVGALAPKYGSNMVASFSNYGKINVDVFAPGASVYSTVPFKDQYDTKGGTSMAAPAVAGVVALVRSYFPNLTAAQVKDVILSSGLPINTKVVVGGDANDVRPFGDLSKSEKMANAYNALIVASKIK

>12. Line 8338: /product="M28 family peptidase"MKHITLLVLFVTLWGCKKEYTYTNEIKEDVVFLSDDKLEGRETGTQGEQEAANYIVKRFKEMGLTPKGTENYFQPFNFKPKTNPHEEVKFVTMEADGTITGTNVLGYIDNQAEQTVIIGAHYDHLGFGSEGSLYRGEEKQIHNGADDNASGVAVMLDLAGKLQKANTSNNYLFMAFSGEEMGLLGSNYFAKNSTIDLEHVNYMINMDMVGRLKQDSTLAVYGVGTSPRFKQVLSATNKSFKIIENESGIGPSDHTSFYLQDIPVLHFFTGQHEDYHKPSDDFDKLNYDGMNLVSNYIFDVISELDSQGKMAFRKTKNESEETPRFKVGMGVIPDYLFDGKGMRIDGVSEGKPAKRAGLEKGDIVIKMGDSTIVDMMSYMRALAAFKQGDTTKVVVTRNGTELEKELIF

>13. Line 8672: /product="S9 family peptidase"

MDKTSTMKYPETKKGDSKSTYFGAEILDPYRWLEDDQSKDTKDWVKAQNEVTNQYLNQIDYRNTIAKKLKGLWDYEKVGAPFQEGNETYYYKNNGVQNHSVLYRKREVGDDEVFLDPNSFSKDGTKSLSQISFTKDGSLAAYSVSEGGSDWRKVVVIDTQTKEQIGDSLVDIKFSQLSWKGNEGFYYSSYDKPKGSELSSKTDQHKVYYHKIGTKQRQDLKIYGFTAEEKHRYIYATVTKDNRYLILTPRVSTSGNKLFLKDLQNEATPLLTVLDHCDSDTYLLDNNETHLFLYTNLKAPNGKIVMVDARNPTPENWIDLIPETEYVLTPSKGAGFIFAEYMTDAICSIKQYQYNGVLVRTIDLPGKGSTTPISGKENQKLLYYSFTNYKTPNSIYEFDPTSGKSSLYWKPKIDFNSSDYTSEQVFYRSKDGTKIPMIITYKKGLKRHGKNPTMLYGYGGFNISLTPSFSVVNAVWLDLGGIYAVPNLRGGGEYGKNWHISGTQRQKQNVFDDFIAAAEYLIEKQYTASEFLAISGRSNGGLLVGTTMTQRPDLMKVALPGVGVLDMLRYHTFTAGAGWAYDYGTVEDSEAMFNYLLGYSPVHNVKNNKIYPATLITTADHDDRVVPAHSYKFAAELQQKQTGENPVLIRIDINAGHGEGKPISLQMQEWADIYGFTLFNMGYKELPAEVHEKIKG

>14.Line 8719: /product="isoaspartyl peptidase/L-asparaginase"

MKSIAPIFLVFFWLLSCHQSQNQGKIDQGFSSLEKSETKPQSFAIVIHGGAGTILRENMSAEQEKQYRDKLEEAIKVGHQILKDGGSSLDAVERTINVLEDSPLFNAGKGAVLTNEGTAELDASIMDGKTLNAGASAGTKTVKNPISLARVIMEKSPHVMMASQGADIYANEQGLSTVEPSYFIVENRVQSLKRLQEAEKKKKSSQKQGFYDSKIKDSKFGTVGCVALDKNGNLAAGTSTGGMMNKRWGRVGDSPIIGAGTYANNGTCGVSCTGWGEFFIRGMVAHDISALMAYKGMTLKEAAKEVIQKKVPEMGGDGGLIAIDKTGEVVMEFNTAGMFRASMNDQGDLYIGMFSE

>15. Line 11301:/product="lipoprotein signal peptidase"MSLKKSLVFIILILLVDQISKIYIKTHFVLGEDVAIFKWFKIYFVENDGMAWGTKISDFIPFMSDRVAKLSLTIFRIVAILGIGYWLYDVTRKRTSKIMILAVALIFAGALGNILDSVFYGILFSDSYGQVATFLPQEGGYESMFHGKVVDMLYFPIWKGYLPEWIPYYGGQFFTFFEPVFNVADVAISTGFGMLIVFNRQAFSKN

>16. Line 11388:/product="peptidase"

MEKKIIGRTDVADFPKLGLFQVDVKIDTGAYTSAIHCSQIEEKDQKLYCTFYSAGHPNFSGKQVIFDSYSKTDVKSSNGFKENRFKVKSSIILFGKTYTINLTLSTRDDMKFPVLLGRQFLNKKFLVDVSQQNLSQNQCAHEHCHSV

>17. Line 11530:/product="peptidoglycan DD-metalloendopeptidase family

MDQRRIEALGRKCLTFLTAALLVTSCGKKKESLPVEETEEIAIVEPEDTFEFGFNLNDYIVKRDTVRSGDSFGVIMERNRLGYPKIFQIAEGAKSSFDITKLQIGKPYTLLCSKDTLQEPKCFIYQPNKEEYVVVNFQDSIHAYTNRKPVTYVEKEVAGVITSSISETLEDLGISPRLTFKLADDIYAWTIDFRRLQKGDRFKVIYTDKYIDDSIYAGIADVKAAFFEHNGEPFYAFEYETDSIKGLSDYFNEEANNLRRAFLKAPVEYKRISSRYNLRRRIALYGNKIRPHKGTDFAADIGTPIRATANGTVIKSAYTRGNGNYVKIKHNATYSTQYLHMKKQNVKVGQFVKQGDVIGWVGMTGNTSGPHVCYRFWKNGVQVDPFRQKLPAAEPIADSLKTSYLAFIQPLKEQLDRIPFKEVPAEEDVLFENSDENLISQAH

>18. Line 12175:/product="aminopeptidase P family protein"

MKYLPIDSQLFVRNRKKFTSEMKPNSLAVFNSNDIYPIGADSTMPFQQDRNIFYLSGVDQEESILVLFPDCPKENHREILFLRETNDHIAVWEGEKLTKEKALETSGIKTVYWLSEFERIFKELMSQSETVYINTNEHYRASIETQTREDRFNKWLKEKFPAHSEAKSNPILQRLRSVKDPVEIALIQEACDITEKAFRRILGFVKPGVWEYEIEAEMMHEFLRNRSKGFAYTPIIASGNNANVLHYIENNKQCQNGDLILFDTAAEYANYSSDLSRTIPVNGKFTDRQKAVYNAVLRVKDEATKLLVPGTLWADYHVEVGKIMTSELLGLGLLDKADVQNENPDWPAYKKYFMHGTSHHMGLDTHDYGILTEPMKANMVFTVEPGIYIPDEGFGIRLEDDVVVQEKGEPLNLMKNIPIEVEEIEALMNS

>19. Line 26710:/product="M3 family metallopeptidase"

MNVLNKPFDNQYHTAPFSKIKNEDFLPAFIQGIADAKKEIDEICENKEAPSFKNTIETLDFSGEQLDRISSIFFNLNSAETNDEIQKIAQEVSPLLSEFSNDITLNKTLFERVKAVYDQKEQLNLTKEQETLLDKKYKSFSRNGANLPEDKKQELRQIDKELSQLKLKFGENVLAETNKYELHITNEEDLAGLPEDAKEEAKQTAEAKKKEGWVITLQYPSYIPFMTYANNRELRKELAIAFGAKGFHGDVLDNQEIVLKIAKLRHRRANLLGYPTHAHFVLEERMAKNPETVTTFLQDLLEKAKPAAEKEFEKLSAFAKELDGIDHLEKWDGAYYSEKLKQKLFDLDDEKLKPYFKLENVINGVFTIAQKLYGLHFEEIDTIDKYHEDVLTYKVTDDNGNYVSIFYADFFPREGKRNGAWMTVYKPQYVKNGINDRPHISIVCNFTKPTKTKPSLLTFNEVTTLFHEFGHALHGMLANTTYPSLSGTSVFWDFVELPSQVLENWCYEKEALELFATHYETGEVIPMDLVEKIKESATFHEGMQTLRQLSFGLLDMSWHGIDPTDISNVKAHELKAFESTKLYPDVAENCMSTSFSHIFQGGYSSGYYSYKWAEVLDADAFEFFKEQGIFSKEVASKFKNNVLSQGGTDDPMVLYKQFRGKEPQPEALLRRAGLIEK

>20. Line 27615:/product="M1 family metallopeptidase"

MKIYKYFFLSMAFLSVQAFAQEQEQEKPETKTGHTNNNKFKQLYDEFSTPNMFRTASGAPGPSYYQQQADYKMDITLDDDKATISGFETITYHNNSPDELTYLWVQLDQNMRAKDSKTPLIEGSGVAPAQQPSGFVSTYMKEPFDGGFNIMEVKEAGGKALQHTINRTMMRVELPKPLKSGEKFSFSIKWWYNINNHVDGRGRSGYEYFEKDDNRAYVIAQFYPRMAVYNDVEGWQNSQFWGRDEFALPFGDFEVNITVPADHILDGTGELVNRKDVFTKEMMNRYEKAKKSYDEPVIIVTQAEAEAAEKNKSTKTKTWKLHAKNVRDFAFTTSRKYIWDMMAVNIGGKDIMAVSLYPKEGNPLWEDWSTKAVASTLKSYSRMTFDYPYHKAISVHAKNQGMEYPMICWNYGRPDENGNYSDRTKYGMMSVIIHEVGHNFFPMIVNSDERQWTWMDEGLNTFTQYVAEQDFGKWYPDALSEGDETYPSRRGPAAKIIPYMGGNQDFIAPIMTKGLNTYQFGSNAYSKPATALNILRTTIMGEELFDYAYRTYAQRWMFKHPTPEDFFRTMEDASAVDLDWFWRGWFYTTDWVDIGVKDVKKYYVSSEPNKYVKDIVAQRGMKMSDLPPLVYMVEEGSEDYKESLKNGSAVENSSTLKEYLMDNFTADERKNINEPKYFYSITFEKPGGLVMPIIVEYTYSDGTSKTETYPAQIWRYNDKEVSKSVASDKEIVKITVDPNLETADIDTSNNSWPKEVQQSDFDKFKNQVRD

>21. Line 27641: /product="peptidase E"

MKLKYLIILFVVPLMAFSSLHKYYVSITQVEYVPEKKSLQLISRIFIDDLEDVLQARYDENLVMAYKNEDKQVDLYVEKYIRSKFGIKINGQEVPLNYIGKEYEDDIAIVYLEVENVKKISSIEITNSVLYDLYEEQQNIIRTKINSKNKSFILTRENDKAMLNYD

>22. Line 27657:/product="carboxypeptidase-like regulatory

MGKIDVIFFIVIFLSANVLFSQEVLLSGRVTSNEDVENIHVINKTSSRFTITNEKGEFQISVKLHDTILLSSLLHHPKEFVVYQKMMEEGIVEVSLTKTTYELNEVVLGKILTGDLGDDVSAVEGEPITARKLGIPSYEGPPLTQSERRLKEATTGGGFVPLNPILNAISGRTKMLKNRIKLERRDDLMYEIRRSLEDKLFEMEDLPEERRNEFFYFCSEDDIFIQRCKVSGDIEILEMLLVKLQEFKKQWQTKTD

>23. Line 27694:/product="dipeptidase PepE"

MKQIIMASTSTIHGSGYLEYLHPELEVFFKNITNILFIPYARPGGISHDAYTEIAQKGFSKIGKNITGIHTYNNPIEAVKNAEAIFTGGGNTFVLVNQLYKNQLIEPIRDALFKGTKYLGTSAGSNICGLTMNTTNDMPIVYPPSFKTLGMVPFNLNPHYLDPVPGDKHMGETRETRIKEFHQFNTQPVVGLREGSWLHIENENITLKGELTGRVFECGKTPYEIETGSSLNNLK

>24. Line 27826:/product="type I methionyl aminopeptidase"MIIVKTREEIELMRQSALVVSKTLGMLAKEVKPGVTTLQLDKMAEEFIRDHGAVPGFLGLYGCPSTLLCSVNEAVVHGLPTDKPLEDGDIVSIDCGALMNEFYGDHAYTFEIGEIAPETKKLLDITKESLYVGIREFKNGNRVGDVGFAIQKFCENEGYGVVRELVGHGLGKKMHEDPEMPNYGKRGRGKKFIEGQVVAIEPMINMGTHKVKQLKDGWTIVTLDGKPSAHFEHDVALVDGKPEILSTFAYIYEALGIESDEETEFRQKALVL

>25. Line 29734:/product="transpeptidase family protein"

MFIFALLVVFKLFSIQFINGEKYRALAESRLVKQVVIPANRGNVYSVGGNLLATSIPKYDIRIDAVTPSAKTFEKYLKPLCDSLSKYSGRPSSYYQKNIRNARANKNRYYLLARNIGYSDYLRLRNFPMLNLGAYKGGLIVEQTTKREYPMGGIAHRSIGYERFDENGNVTRAGIDGAFGAKYLRGTDGKRLKQKIGKGEWKPMTDYNQVEPKDGYDIYTTISTNIQDIAHHSLLQQLEYYEADHGCVIVMEVKTGEIRAISNLAKTSEGTYYEKRNYAVWEALEPGSTFKTMALMAALEDKLIDTSTVVDTKKGKKFFYGRPINDSHHGGFGKISAARALEVSSNIGLATILDEGYKDNPEKFINRLKDWKLNEPLGLPINGEGTPDIPEPGSKKWSRNALPSMAYGYNLELTPLHTLTFYNAIANDGIMVKPRFIREVKELNKSMESFEREIINDKICSDKTLKEVQDILKHVVERGTGSSLYSPYFSMAGKTGTAQSEYWMEGWKENKRYVSSFAGYFPAENPKYSCIVVIHKPSIKKGYYGADVSGPVFKRIAQKIFTDTPIIDRMGTLEVNDPKVGSEYESFFKLAQNHKTIMPDVTGLPLMDAISLLENMDIGVKVQVEGNGVVKSQSIGKGEKLKQNQTIIIKAS

>26. Line 29962:/product="C40 family peptidase"

MQRILTFLFAFMLLASCGSSKKAKVVTKRDKKTSSSTSSAKSDNWSTNAESTSTADYSTIKKIIKEAESYRGTRYKYGGTTKKGMDCSGLVTTAFKSENIALPRSTGELSSYGDWVDLKEVKEGDLLFFATSKNSRKVNHVGLVVDARPGFVQFIHSTTSAGVITSQLSERYWYFAFVQARRVM

>27. Line 30064:/product="S9 family peptidase"

MRKFFLFYFLATFLISAQNKEITLEDIWTGTFRTERMDALHSMANGQQYSVLNFNRNTASTSIEVYDYQTLEKVRTLVDSKDLDQVRYFTDYTFSEDESQILLATEMQAIYRHSALGKYYVYNVETGALTLVSEDKIQEPTFSPSGDKIVYGKGNNLFVKDLKTGETTQFTFDGEKNKIINGITDWVYEEEFAFVRAFDWNADGDKIAFIKFDETEVPEFTMDVYGMDLYPHEDTFKYPKAGEANSQVSLHLYDLNADKVSEIEVNKSYTDFYIPRIKWSQDADVLSAQYLNRHQNELDLWMIDTKKGNPELVLEERDGAYVDVTDNLTFLKDNSFIWTSEKDGFNHIYHYDKHGKLVNQVTKGDWEVTNYYGYDEKEKKIFYQSVENGSINRDVYSVKLNGRDKERLTKSEGTNNASFSADFSFFINTFSSATTPPEYTLNDAKNGNVIKSIKDNDKLAEKIQDYTTSQKEFSTIQVNGEELNMWMIKPANFDENKQYPLFMYQYSGPGSQTVSNSWNSSNDYWFQYLAQKGYIIACVDGRGTGFKGAEFKKVTQKQLGKYEVEDQIAAAKKMGERTYIDQSRIGIWGWSYGGFMSSNALFKGNDVFKMAIAVAPVTSWRFYDSVYTERYMTTPSENPKGYDENSPLSHVDKLKGDFLLIHGSADDNVHLQNTMRMVEALIQADKQFEWMIYPDKNHGIYGGNTRLHLYKKMSHFIDKTLGDKIQKEDLEQEEKSEIKG

>28. Line 31959:/product="dipeptidase"

MKDISNYIETHQNRFISELVDLLKMPSISADSAYKKDVLNTAEAVKKSLLDAGCDLAEICETPGNPIVYGEKIIDKNLPTVLVYGHYDVQPADPIDLWTSPPFDPVIKKTDLHPEGAIFARGACDDKGQMYMHVKALEFMTKTDQLPCNVKFMIEGEEEVGSENLAWFVPRNLEKLANDVILISDTGMIAKDVPSITTGLRGLSYVEVEVTGPNRDLHSGLYGGAVANPINVLSKMIASLHDENNHITIPGFYDKVEELSTEERAEMAKAPFSLEAYKKSIDIDAVYGEEGFTTNERNSIRPTLDVNGIWGGYIGEGAKTVLPSKAYAKISMRLVPHQDWHEITDLFTKHFESIAPQGVRVKVKPHHGGQGYVTPIDSMGYQAASKAYHETFGKTPIPQRSGGSIPIVALFEEQLKSKTILMGFGLDSDDIHSPNEHFGVWNYLKGIETIPWFYKYFRELSE

>29. Line 31999:/product="M20 family dipeptidase"

MQLSNSEEELMNHLWKLEKAFMKDLLDSYDDPKPAPTTLATLLKRIKDKGFIDYKVYGKSREYYPLVKKKDYFSKQVNGLIKNFFNDSASQFASFFTKETNLTKEELEDLKALIDNEIKKK

>30. Line 32024:/product="M56 family metallopeptidase"

MLIYLLKSSGCLLVLMLFYKLILERESIHQFKRFYLLGSLVAAFLIPLVTWTTYVEIPLEPEGVPFDPTLLPMEDMPTDEPFNWAFYLGVIYGAGVLFFGLRFINNLWSLHLKIKRNQKIPREAYIQVLILESIVPHTFLKYVFVEKSQFDSKNIPDEILLHEQIHVEQKHTLDILLIEVMQILLWFHPLIYLIKKDIKLNHEFLADQAVLSHGFETKNYQNILLDYSISSSHNQLVSSINYSLIKKRLKVMKTHTSKQVKWLKSLVLLPLLALLIYSFSSRKEVFHSNLIQNQLTIENKKSDIELQVDETGKLYYQSKEITIEELSQITNIEDHIQIKLVFPESRPEKTIKKVNKQITDFIRSKDVKHFSICSSFMENDQKTSNTVIRNLDIPELQVEQNPTRLILNGKSTNFDKLLDDFKQITNNQKSDLYINTKGENLRYDVLMQIANSLRNQFGEITVSDGVIRDPNNSSLTKNPNGSVSIHSLQEGASKAQIAEYNKLAKKYNSQPKEEQRFYKKDVERLGYLYGLMTKEQKAHAEPFPNIPPPPPTPPVEMGTMSPELQKLYKHYTKEADAYVKAVSVYRKTKKGTQEDLKNQYSDLMKLYKTYYELAYKEKLTPVLPPPPPPAPEAPEVIEVIEVEGYSKSKVKKAPKTADLQEKEVIGYPSPESRKKSAAIYAKENPEKVSKRKTNSGEVIEIVEVPVNKLDTNTPKTAYDFVKSHKNDDITYYYNDKKVTYDEILKIVKKEPNININTNINDHKGTVKFWSKK

>31. Line 32489: /product="zinc-dependent peptidase"

MLQILLEEEENIERIGKQVFFMLYILGMATFLSVRFFRFLEMAYVEYIDKKLFYTHLNVGLKKLQPHQQVILKRKFRFYQYLTPKQQSFFEHRVARFLREKEFVGKGLEVTDEMQVLVAATAVKMMFGLRDYRLQIIQTIFIYPEAYYSRLNDAYHKGEFNPQKNLLVFSWEDFKDGYQIDDDNINLAIHEIVHAIHFNFLSRRGKSASAGIFLNSHAELVSYLNNHPKVLEEMQSSGYFRAYGFTNDFELLAVIMENFIETPEMFKREFPHIYRLVKQMLNFHFKGY

>32. Line 33335: /product="M13 family metallopeptidase"

MKNVILFSALLLSLFSCKEKEAQTKIDEEQTSGINLSHIDSTTVPGNNFFRYANGEWLKTADMYPGMPMNGNMIELTFRTQNQVGEIMAEVLKEGDFPKNSPKGQIQAFYKSYMDTITRNELGMKALQPELDTIFAAKTRSDLSRIMAQPLMPHMWGGYVFLDAKNTKRYVPIISQPDLGIGEMEYYLQDTERNIDIRKKYVDLIKSLFTYAGIDSPQKRAEAILRLETKMANVFWTGTQKRDPDKMYHLMTTEELKAYAPGIDWQAYWDANGKGSYSELVVQTDTAIKGVSKIFGEASMDDLKSLMAFYLIKSNATFLSTEINDVYFDFAGKIMMGVAEQQSLEDKAKGILNKFLSWQLGELYADKYYTTEVEETIEMLVGYMKQAIVNRLESNVWMDEETKKEAVAKFNNIHWKVGRPDKIIDLSSLEFEEDDLIGNFRKIAKSESEDQISRLTEAKREWEWLMPPQMVNAYYQSEMNDVVIPVGILQSPNFDPTADPASNFGGVLSVVGHEVGHGFDDKGSKSDAEGLLRNWWTVASREEFENRATELANQYDQYETIPGVYLNGRLTLGENIGDVGGLAIALEAYHNYVNDHQNGEAPVIDGLTGDQRFFIANAQLWSWIMTDDYARMMAVSDNHSPGKFRVNGVVRNMDAWYEAFGIKPGDSLYLAPDSRVKIW

>33. Line 34632: /product="zinc metallopeptidase"

MLGYYILLGAIALVSWLVSNQLKQKFHKYSQVHLRNGMSGREIAEKMLADNGIRDVQVISTPGQLTDHYNPKNKTVNLSESVYSQRNAAAAAVAAHECGHAVQHAQAYSALGMRSALVPIVSVTSGMSQWLVIGGLVLGAGAGMGLGYWIAVAGLAFMGFATLFSFITLPVEYDASNRALAWLKNKNMLSQEEYAGAEDALKWAARTYLVAAIGALASLLYWAFQVFGRRD

>34. Line 46474: /product="carboxypeptidase-like regulatory

MTFTTIKSLLIYFILLFGATSFAQSISGKVLSDKEEPISYATVQIGEHFGVITNEEGSFSINTSGFEPTDSVKISCMGYEKIGMLLNEFESQDYHLIEKVNELSEVYLTNRMLTLDSILYYMDKNLDNNYKTKMVNYNLFRRTTQYIQGKNVNLEIDKSTGFKKKQLEAFNKEFQELGNSLLNNRSKQYTEFVADLSVLDEKNSKMTIDKAMRLLDERNDQTIERLAERGKDIVTKHLDTNKVYTVKTGWFKISDSVKLGESSRENNKMEDTINSVGLIRKLTYEMLKEHNFKSGSIFNFVTDQRKYDYELKGISFLDSELVYVVDFKPRRGSADFEGTLYISNQTFAVLKTDYKYFKGRVGEKLNMKFLLGIKYAENGKSGSVIYKKGPDNYYYPKFITEEIDRYFYIDRPFKFKENDGNNKVSFEFLVEGTFKERNELLIMGTNDITNSDYEAIKEEKDIDYETPKQYDPNFWKDYNVLQPLQEMKDFKVDETYTP

>35. Line 46613: /product="M24 family metallopeptidase"

MKTFILSIFSIFIFSEIVAQTDLPTDYLSKEFHRNRREALRASMPENSVAVFFANPVRNRANDVDYVYHQDPNFYYLTGYKEPHAVLVVFSTDQTDKEGNTYNELMYVQEKNPMAEMWTGHRLGIEGAKNKLGLENAYNGKEFLENNIDFSKFDKIMFENFNDDYRNSSRNKADLHDLVQSFKSQINFETFKPSSQMQERAYQMIKSTEIENSANVAQTLGKVLEYYPDLKEDQIISKYAASDNNELRRELKQQVIIIEAENQSNIDVRGLSTLMAQLRQIKTAEELTLLTKAVRISSMGQREVMKAMHPGMSELEVQGIHEYVYKKYGSEYEGYPSIVGAGNNGCVLHYIENSKMHVENELVLMDLGAEYHGYSADVTRTIPANGKFSKEQKAIYDIVYEAQEAGIEASVVGASFQSPNIATRQVVNEGLLKLGIISSIDEVHSYYPHGSSHYLGLDVHDPGTYGNFEANMVITVEPGIYIPDGSPCDEKWWGIAVRIEDDILITEKGPVNLSGEAPRSSAEIEALMKESSALDDFVLPKLD

>36. Line 46955: /product="C40 family peptidase"

MKYGICNLGIVPLRLEPSDKTELVSQVLYGEFFKVLEQRKEWSRIRISYDKYEGWIDNKQYLEILEEDYLKLKSEASILSTDLVEFVQDSNDQLYPIPMGSTLNGLSLLNHKHDGHNFQGKKGKEFLLETAFNYLNAPYLWGGKTPFGIDCSGFTQMVYRLNGYSIFRDASQQATQGEALSFIEESEPGDLAFFDNNEGNIVHVGIIMNDNYIIHAHGKVRIDRIDHSGIFNQDLRRHTHKLRVIKKII

>37. Line 47724: /product="carboxypeptidase regulatory-like

MKNILRLFLILTLIYSCSDDKTTDDLTGFAINGRLLAPNGTDPIPNAMVSARSNNTVVAESFTDVQGQYALSLPKGNYELTFTKGKFSGVLQIDVEGAKTNEDALLDILPTIGVVTGHFDHIEHVLLNIGLVDPITQEPLFDIIEGNNFGRPSGAHGHRESLHMSQNKGMMDEILLEPNVDFNFEDLMNDPALLGSYDILFLNCGLNESLEDMGDVLMDYVYNGGILYATDWAAGYLNDITNGGADYMTFYDPEKSGTSLTTTATLLDTTLSDWLELNFDIVLEDTIEIEEFLSSWQVVDSYDTTTTISWLEGEVSYRDSGNNIITETKDLAFTFLHGDGAVFYSSFHTENYDPEFSDVDRIMEFFVFEMSVIQP

>38. Line 47743: /product="M1 family metallopeptidase"

MIHTFKCYFTCCLVIMGGLLFGYSQTIPADIYVPLEFQKAYQQGTRMANGTVSPKYWQNHSVYNIKATLEPSTRLLTGEAIITYYNESPDSLKSITFHTYHDYYKPDSKKAGFFRAADANEKPHEGMVLGQVLVGGTPIDLKDRKQARYGGTNYSIVLGDNPVPPGGQVNLRVDWHYVIPGEGFERSGAIDPTSMFVAYWYPEMAVYDDIDGWDRVVYDAATEFYHDYSDYHVELTIPDNFMVWASVAPDNPTEVYSEEVQKRLEKASKSTEAVSIIGESDFKTTSTKNLTWKYTAKNFPDFSFALSDHFVWDAAHYSDKMGDYFIQVAYPTSHPEFAAVLETIGMSLEVFHNQFPVYEFPFHYFTIFNGLVGGGMEFAGMANDQETSAEKLKTYGYEVLNDTEAQFGLSLHEMCHMYFPFMMGINEKKYAWMDEGFASFSSFFMDNPFKMPERDLPYLGSQEVLPIMAPSHLFEGSGLNSYTIGSHSYKSLYELLGEETFTKCMKTYMDAWKHKHPTPYDFMMTFNRVSGENLNWFWKAWYFDWGYMDMGIEQVNGQQVTLKNLGGRPMAMTIKATLDDGSVLEEKINPKVWKDSDIFQWKLSSTKGSLKKVELEIPSYDAKASNNTWEKS

>39. Line 50371: /product="carboxypeptidase"

MKKLFFSLLLLVFIIPNAFSQKREIPTDTVITTSHQATINGQNFNYEAKVGTLPVWNKDNEPIATLFYTYYKRSLSKPDDSRPLVFSFNGGPGSASVWMHIGYTGPKILNLDKEGMPVQPYGVKDNPYSILDVADIVYVDPVNTGYSRMVKNKEGKDPDRSQFFGINADIKYLAEWMNTFVSRYNRWNSPKFIIGESYGGTRVMGLAEELQNKQWMYLNGVIMVSPADYKLYNNGLISSAINIPYFTAASWYHNKLPSDLQNRELESILPESEQFAINEVLPALVNGAFLNESRKQQLAEKMAKYTGLDKERILDQNLDLSTFYFWKELLRDDKLIIGRLDSRYLGIDKKDTGDRPEYFPELTSWNHSFTPPINHYLKNDLEFVTDTKYNMFGDVHPWDLTENNTRDRLRQAMAQNPYLNVLVQSGYYDGATTYFQAKLTMGLVDPSGKMKDRLNFKAYKSGHMMYVRDEDLKQSTADLRNFILKSTGHKTSAKYDNPKN

>40. Line 50413: /product="peptidase M61"

MNRILTAVGVGVLMMACGTTNQTTKDLAISNPIESSIDLTKVKDDKVPVIINPGRFTSETEIYRLPRVVQGTYSVSDFGKYIEDFKAFDYEGKEIPTEKIDTNSWQINQASNLDYVTYYVNDTFDVEVTGGIGGDTPFSPAGTNIEPDNYVLNLHGFVGYFDSLKNAQYKLDVTAPSDFVRTSALQVVGTSKSDDGQTMTSSYYAPRYFDITDNPMMYGNLDVEEFQVGDIKIVLSVYSPTKAHTAKKLKETMFKMMKGQKTYLGDINSTPRYDIYLYLSRGDEQSPKGFGALEHHTSTVVVMPESMPEAALDESMIDVVSHEFFHIVTPLSVHSEDVHYFDYDKPTFSKHLWMYEGVTEYFATLFQVDQGLVSDDDFYDKIEEKIKTAAMMNDSMSFTIMSENVLDEPYASQYYNVYQKGALIGMCIDILLREESNGQRGILSLMKELSMKYGKNKPFEDDKIIEEITAMTYPSIGEFLKDHVVGDIPINYNDFFKKVGLAKSKAKVKTNYIQNDGVLIVGANPQDGTIFFSDAVQNNSFWKEQGVQPNDVIKSVGGEQVTLQNANNIFQEVFMWQPGMEFEMVISREGQDIELKGTLEQSFTEGESLRPVEDATESQKKLREAWLRG

>41. Line 50703: /product="M13 family metallopeptidase"

MKNIFVIFIALLSLVACNKTSKIENKPVIEITGIDENLRPGDDFFRYVNAKWYDTVSIPSTQAGVGAYRFMNYPQRIKLKSILDSVSSHEYQEGSIEQIVGDFYASGMDTITIEKRGYDPIKGILNNVENIKDKSEIMKFVAEAMKMNNNSFIGLSVGPDDKNSAMNMAHAYQTGIGLPDRDYYFKTDAATFEIQNAYKEYLASLLQLIGTDKGVAVKQANLVYDLEKQMAKSHKTRVERRDIKANYNKMAVANLNTDQPFIEWSQFLGYLNAQTDSINVAQPAYYETLNTLINSVPLKDWKIYLKAKTLTNYADYLSTPFVDASFEFTKVLSGQAIQKSRGEKMASAVDGYLGEALGQLYVKAYFPESAKSRMLDLVNNVQKAYAARIDKLEWMSDSTKQKAQEKLFAISKKIGYPDVWKDYSKVHIDRDQYFENVLSASANLFQTNLNNLGKPVDKEEWFTTPSTVTAYNNPSANEIVFPAGILQPPYFNNDADDALNYGGIGMVIGHEFTHTFDDQGAQFDKEGNVNNWWTDSDYKKFKGRIEQVIDLYDTFTVLDTLHINGAMTVGENTADIAGIAVAYDAFKMTPQGQDTTKLGGFTPDQRFFLSVAKIWRVKMKDEYLRLWINNNPHSPPVWRVNGPLMNTTPFYEAFDVQPDEAMFLKEEDRITIW

>42. Line 50927: /product="S9 family peptidase"

MELEAHNDVRIDDYYWLNERDNPEVIDYLNAENSYTKKVMEHTEPFQEKLFQEMKSRIKEDDESVPYKLNGYWYITRYETGKDYPIYTRKKETLEAEEEVLFDCNDMAKGHSYFKLGSISVSPDNKLAAFSVDLVSRRQYIIQVKNLETQEIYSTKILNTTGSVTWANDNKTLFYARKDEQTLRSHKIFKHLLHSKVENDKEVFHEKDETYNTYVYKTKSRKYIVIGSSSTLTTEYRILDADNPEGDFKIFSKRTRKLEYAIAHFEDYFYIITNKDKADNFKVMKTTTDKTDKQFWQDVIPHREEVLIEDIDIFKDYLVVSEREQGLNNLRIISWNGEEDYHLPFSSETYTAYVGNNPDFNSDVLRYSYNSLTTPSSVIDYNFKTKTKVVKKEQAVLDSTFEKANYESHRIWATARDGVKIPISLVYHKKFKKAEGNPLLLYAYGSYGSTIDPYFSSIRLSLLDRGFVYAIAHVRGGEYLGREWYETGKLLKKKNTFYDYIDCSKFLLDEKLAAKGHLYAYGGSAGGLLMGVVMNMNPELYQGIIAAVPFVDVVTTMLDDSIPLTTGEYDEWGNPNKKTYYRYMKSYSPYDNVKSQDYPNVLVTTGLHDSQVQYWEPAKWVAKLRDLKTDENILLLYTDMDSGHGGASGRFESLKEVALEYAFLLDLEGIKE

>43. Line 51330: /product="carboxypeptidase-like regulatory

MKHTFFLLIFTITQLAISQERKTGIIKDADTKEPLEFVSVYIDNGPDSNYTGSISNGEGEFSIMDNGSKVTFSYLGYEEKEVELQDGFNEIFLKPKDFVLDEVVISDVSAEDYLKSIIKAADNKIDKNTLFKSYCREIVKVNDEYTKFSDGLVNYYVKKGNGKSVITLPQHRAFKSDKISEEDFGSIDNINSAFTLDEYVKNAYNYKFIKKLLKDKNYDFVRKIKKEEGGLEYEYIEVSPKSDVEELLYKGYVVIDPKTGDVLELKFYTSSDHLKYSKLQNVMLIMKVKVNSLLIWTKFRVIEDKYVLSYNQKSVGMYLKFGKYVDDNFNFSSDLFVYAFENDVEIPDDGYDNRTIYEAGTSFTENFWENYNAFPLNNEEEAFIESVSK

>44. Line 52068: /product="aminopeptidase"

MKIYTFLTLILMGSFSLYSQKYEFTPIIDLDATEVVSQGITGTCWSFSTTSFLESEIIRKTGKFIDLSEMYSVRQTYPKKAWNYLMRQGNAQFSQGGLAHDVINSARDAGLVPQEVYTGYLNGQTKYNHIELEEVLKNVLDTYIENPANTLSPTWKTVVEGILDSYLGKQVESFTFEGTSYTPKSFMEMVQINPDDYISFTSFTHQPYNSKFILNIPDNFSNGSFYNLPLDDLMQVITHALENGYTVELDCDVSEKTFSAKHGVAVIPEDESMNKEILTEIRSEKTITPEFRQQEFENFHTTDDHLMHITGLVKDQKGNTYFKVKNSWGTDESRVTYGGYVYFSEAYMRLKAISILIHKEALPKGIKQRLSIQ

>45. Line 53604: /product="M28 family peptidase"

MKAFLIASILTLIGSCATQKYSDKIDILKNGIHIKDSTAILEFSQTITAPELKVHLEFLANKELYGRETGEAGQKQASEFLKSYYQSLNIPSPMEDSIYFQKIPKDYFPEKYNASENVLAFIKGTEKPEEVVIVSAHYDHLGLQDGKIFYGADDNSSGTSAILEIAEAFQLAKEKNQGPKRSVLFLHVTAEEIGLQGSKYYTEHPAFPLDQTIADLNIDMIGRVDPSHEDNPNYLYLIGSDRLSRELHYISEKINNSFFNFSLDYKYNDESDPNRYYYRSDHYNFAKNNIPVIFYFNGEHEDYHQPTDTPDKINYELLEKRARFIFATTWQLANMENRLLMNEDF

>46. Line 53623: /product="M28 family peptidase"

MRKLILSILVAQLFWACGSQKTNTSSSEPSVPLADETTYAATITSQELKDHLYTYASDEFEGRETGAPGQKMAVNYIREEYKKLGIKGGMPDGTYFQEVPLEVLDTPEVNLTINGQEFKITDNFISLSSAMDGSLNANEVVFAGYGIEDSNYSDYSQLDVRDKVVLIKSGEPKTENGTYIISGDSTPSKWSNFRQEFASKRDLAKEKGAKCVFYYNPQLYEMAKGRFGSNRRKSMSLKGKSGDMFYFMVNSDLAQAILPEIDQDETAKVIPAKLSLDYKNVSEEFSSENVLAYIPGSEKPNEFIVISAHLDHEGIKDGKVYNGADDDGSGTVAVLEIAEAFQTAVKNGQGPKRSILFLNVTGEEKGLLGSRYYTDFDPVYPLANTVANLNIDMIGRVDPKHDDTDGNYVYLIGSDKLSTELHNISEEVNKKYLNIAFDYTFNDDNDPNRFYYRSDHYNFAKNNIPVIFYFNGTHEDYHQPSDTPDKIRYDLLEKRTRLVFYTAWEIANRPNRIVVDKAE

>47. Line 55175: /product="peptidase"

MKRFFKVYIIALSILLSSCGSLLNPTVPITNDIQFTVNLLDRSNDTFKVTVTPPSLGEENDIFNFAATSPNTYQIMDIGKYVTSFRAFDKHGNEIATKHISTNQFKISKPHKVIRIEYEISETWDSPVDQNPIHLRFGTSIENDHALINGQAVFGYFKGKKKTPIKLQLQHPAEWQVATSLKKSSSGKYLAKNYSQVVDSPILLGYLTRESMTVQGAVIDIFSYSKTGVVTASQIMESLKKMFETAGNYIEGIPVDQYTFLFMFEDKTDGSWAHNRSSGYVYRETPWKNLEKEILDLAAHEFFHIVTPLNLHSEIVSKFQYDTPLPSKHLWLYEGVTEWASKMMMFRGGETTADEYLSELEKKVYISKNLYNNHYSLLDLALTSYTSDGQKQFGNINMKGALAASLLDIRLIELSKGNTGLMDVIIKLSRKYGPNKSFNEDSFFADFTEETYPEINDFFDKYIIHAEPLPLQEYYFKIGILYDEDTNKFTVLKEPSEEQLKLRNKWFEPLRKRGISLFLPQTSFINYVGTMDSTIESLYEVISGERGQERNWEFFKYLFKPDAKLIPTARAQDSTYKVKYISPNDYVKSAETWMMDNGFFEKEINRVVNTYGNIAQVFSTYETFRTQTDVIPFMRGVNSIQLLFDGKRWWIVNIYWTQETPENPIPPEYLPRVD

>48. Line 56330: /product="zinc metallopeptidase"

MKWKGRRQSSNVEDRRGQSSGGSGLGGLNPMLLISLVRFLFTKTGLIIAGVLLVGSMLLGYNPLNFIGQLFTGSPVTSETSAPYQGTQKEEELADFSATILASTEDVWNQLISNYEEPTLVLFTGSVSSACGYASSATGPFYCPGDDKLYLDLSFFDDMERKLNAPGDFAQAYVIAHEVGHHVQNLLGISDKVQRLRGQMSQTEFNKYSVRLELQADFLAGVWAHHSQQMTQMMETGDLQEALNAAYAIGDDRLQQQSSGRVVPDSFTHGTSEQRIRWFKKGFETGDINQGDTFNANPL

>49. Line 69502: /product="carboxypeptidase-like regulatory

MKRLILFLVIGMIGFIGHSQIEIKNKVVDFQSFLPLENCSVYVKNTTIGTVTNEDGKFVLLIPEKHQTDTLIVSNIGYKSYKIPVNEFDDSMEIILEEDIASLNEVLLVADTRPETANEIIERALERLPRNLPESPYLQKGFIRHKERNKREFKWLIESAITLYDSSFPSGANENIKINVDQVRKSYDLRDVDSLLVYTSYLKNHVRNFKMKSRNLKRDTIRTSSLVKAIRWNDGRVNGLENLFKGKLNLVRNSHDPKALFGENMLESHQFELDTILVDNERKIYKIKIEEGNDFVDLHTKGIFNNGFQAQGWIYVYWDNYAIKKIEYELVAASDAQKSRSKTLFDTYLNHKLELSYMEYDERMYLNYIYYETPKLVNVGDKSNQDNANNDERYYYTVQEVLFTEIVVDPEEVQEQIVGKSWDADMFSPKPYDKSFWKSYNTLLESEEEEKLIEDLTQKYSLYKE

>50. Line 70078: /product="M15 family metallopeptidase

MKYTISVLIFVIFGFFSTQKEPLPEGFVYVDEIIPDLEVELRYYSTHNFVGDTIAGYHANRLILTLETAEALKMVQEDLENENLCLKVYDGYRPQQAVNHFVHWARELNDTINKQEFYPEVKKGNLFKEGYIASKSGHSRGSTVDLTIIDAETLEPLDMGSPYDFFGVQSWVSYQSISAKQKENRLRLQRVMQKHGFRSYSKEWWHFTLRNEPFPDTYFNFSIN

>51. Line 70095: /product="M14 family metallopeptidase"

MLCLWFKYGFCQENFVTQFEKSNGLETAAYYDVIAYYQNLATAYPTITVDSMGITDSGERLHLVTFDPERNFDFDSNRKNKRILLINNGIHPGEPDGIDATMMLFRDYASGQLKAPKNTIIATIPIYNIGGSLNRNSGTRANQNGPKEYGFRGNSRNYDLNRDFIKCDTENAKSFAEIYHLVNPDVFIDNHVSNGADYQYVLTHLFTQHNKMGGPMGAFIHETFRPQLEQELSSKSWDITPYVNVFNAVPEKGFSQFMDYPRYSTGYTTLWGTLGMMLETHMLKPYKQRVEGTYLFMQTVVNLTETHGEKIRVFKEQQTTKYKPNESYPLDYKIDTTRFSTLEFKGYEGEIITSEVTGLSRLKYHRDQPFSKQVNYQNYFVPSVEVDIPKAYIIPQGWHHVIELLKLNQIEMTQIQKDTTISVESYRIQSFETRNSPYEGHYLHYNTQILSSKVEKTFRAGDFIINTQQKGIRYLLETFEPQAPDSFFNWNFFDTILQQKEGFSPYVWEDKASEMLKQNPDLKREFENKKYTDGNFAKNWYAQLEWLFQKSDNYEAAHMNYPIYRIN

>52. Line 70291: /product="carboxypeptidase-like regulatory

MKKCILLIVLLSFGLNFAQTKIIDKKTNDPVSFATISFGDGFGVFANDQGRFTFTKKLYPDIDTLYISALGYKDLSLPAEPLPETIFMEEEIDHLDEVVIYANLEDRKYKVEKLKPYLDDDYYSCWLPTLESEIAVFFKNEGSELKQLTSVQFPIALESEDWNKRKRSNADKKEFSTLFKAQFYKNEGGYPGDPLTYQQVTFRVTEEDGDQYDLDVTSNQIFIPENGLFISIQVMGYTDEQGNLLPNKQYKEIKSRSGMVKIPTNFRPLLPFTDEIRETRTFIKRVFQYTNEWSPFQKGSFQGSTLLKAGKNNYGMGISYRKYKDE

>53. Line 70744: /product="carboxypeptidase-like regulatory

MKNQINLNIKTPCQEDFNSFSKTPNGKFCQSCKHEVVDFTTMDHKEISLYFSSKENNNTCGRFKQSQLAAFTIPVSKTKINFFKGIGLACISFFSLHNLQAQDVIKQTDPMNNSTNIQNQVANSPVKVSGMVTEDSIPLPGVNILLQETNIGTTSNFDGYFEFPKKLKKGDVLLFSFIGMESQKVVIEDESSPQLELKVNMNSDSYILMGKVAVKEVYKSK

>54. Line 71252: /product="carboxypeptidase-like regulatory

MKTLRVTSHFPKSLLLLAAFLLSACYFNVSAFSTENTTLIQQNYDEYKGSVYDAKTKDPLTFADITLKGTNISTITNTEGEFMLKVPKDQSNGIIEISFLGYTKKELSLTEFKNNSKIYLEPSVSELSEVVINRPETALALVEATLKKKGENYINQQNIMTGFYRETIKKRRKNASLSEAVVEIYKQPYNSNKKDKVKVIKSRKDVDYSRLDTLALKLQGGPFSTLYVDMIKYNEFIFDFDTMKYYDFSFDNSTQINDQNVFVVNFKQKPEVDSPLYYGKLFIDTENYALVSAVYNLNVEDEKEASKLFVKKKPARVDVYPKEAAYRVDYRTHNGKWHYRYSNVQLMFVVDWKGKLFNSRYSLNSEMAITDWKEDASKRLSSGESFKPSMILMEEASGFSDPEFWGEYNIIEPEKSIESAIEKISKKMAKN

>55. Line 71510: /product="S8 family serine peptidase"

MMRKITLFIFCIFSISNFAQQDAWVYFVDKENVTESLANPISILTQRAIDRKAAHGVLIDTRDVPVTESYISEIKNQDGITVLAKSKWMNCVHVRGEFEAISELLSLEIVDEIVYADGSLNARPGEISNKFETEEVMVDFVYGNSGNQIEMFNGDVLHEMDFTGAGMVIAVIDAGFPNVDTMDGFDRLRTNNGILGGYDFYDRNEDVYANTSSSHGTLVLSDMAGYVEDQFVGTAPDAEYYLFRTENAPNENPVEESLWVEALERADSLGVDLVNSSLGYKDYYDNAAYNYSSEEMDGETAFITQGANIGYEKGLLIVNSAGNAGENGVNAPADSEFVFSIGAVNSSGVYALFSSQGSDFQPVIKPDVAAQGQSSTVINSNDQIVTASGTSFSSPILAGGIACLWQALPDLDNGEIMQLVRESASQFDSPDYLLGYGIPDLSLALNLALSTENNTINLPEVKVYPNPVKANLNIYLPDEWASATIKLYNVLGKMVIQQKLTNTNNSINVKHLDRGIYLLQLESGNYHKTIKLVKD

>56. Line 71761: /product="peptidoglycan DD-metalloendopeptidase family

MDPNTFNSFLSQISPEPLRVIDDTIPLSSYVSIDISENQAQLHQFDVTSSKDWEVFINDYLKKHEAQVAYGGYLETRDIYKRSKHFNKSDKETERNIHLGMDLWAAEETAIFAPLDGKIHSFRDNIQFGDYGPTIILEHHIQGIRFFTLYGHLTRESITNLEVGQSFSKGQIIAALGAPEVNGDYAPHLHFQIIRDMQAYYGDFPGVCSKKNLDKFKELCPDPNLLLKVFNGTPIF

>57. Line 73371: /product="peptidase T"

MLNKQHIIDRFTKYVTIDTESDPDSKTTPSTEKQWDLANLLAEELKAIGMTDVSIDENAYIMATLPSNVDHEVPAIGFISHFDTSPDFTGANVKPRIIENYDGKDITLNEEENIILSPDYFDDLLQYKGQTLIVTDGTTLLGADDKAGICEIVSAMEYLINHPEIKHGDIKVGFTPDEEIGRGAHKFDVEKFGADWAYTMDGSQIGELEYENFNAAGATVKVKGKIVHPGYAKGKLVNSMYIATEFINSLPRLETPEHTEGYQGFFHLHDMEGHVEETVLKYIIRDHDKNHFEARKEVMSKLATEINQQYGKEVIEIEIKDQYFNMKEKVEPVMHIVDIAEEAMKQLDITPIIKPIRGGTDGSQLSYMGLPCPNIFAGGHNFHGRYEYVPVESIMKATEVICKIAEITAEKHK

>58. Line 73596: /product="carboxypeptidase-like regulatory

MKQLILLITLCFCSVVVFAQEPTTVLGKVINATDGSTMEKVNIVNINQVKGTATNGEGKFEIQAKANDTLYFSYLGFKSIKVRVTNDWLKYGESTIEMTELAFALEEVVINQFNLTGVLEVDIKQVPINNNYRYSISGLPSTGYEAGRSSNAVTRVLGAVFNPADFLYSVFGKEPNEMKKLKKMKQDDEIRNLLANRFDREMLTALLQVDRVDLDEIVRQCNYSKGFIETANDLQILDAISQCYEEYKVLSRTKRSGRL

>59. Line 82923: /product="M48 family metallopeptidase"

MTASSLLYIILAIIIIDFIIDKILDGLNAKHFNDHLPDELKDVYDPEEYKKSQHYKATNYKFGLLSSTFSLILTVGFILLDGFEIVDNLARTLTDNDIFVALIFFGIIMLASSIITIPFSYYKTFVIEEAFGFNKTTKKTFFLDKIKGWLMSAVLGGGILALIIWFYKSTGNYFWFYAWGIVTVFTVFMNMFYSRLIVPLFNKQTPLEEGSLRNKISEYAESVGFHLNKIFVIDGSKRSTKANAYFSGFGSEKRVTLYDTLINDLDEDEIVSVLAHEVGHYKKKHIIFNLITSILLTGFTFYILSLFISNPLLSEALGVEKHSFHIALVAFGLLYSPISEITGLIMNWFSRKFEYQADDYAKNTFRAAPLITSLKKLSKNSLSNLTPHSSYVFVHYSHPTLLQRIKNLKK

>60. Line 82979: /product="aminoacyl-histidine dipeptidase"

MNSDIKSLVPQELWQNFAKLNAVPRASKKEEKVIAFMKAFGEKLGLEAFEDEVGNVIIKKPATSGMEDRKTIVMQSHLDMVHQKNNDTQFNFDTQGIEMFVDGDWVRAKGTTLGADNGLGVATIMAILESKDIPHPAIEALFTIDEETGMTGAMGLKGGLLHGEILLNLDTEEDDEIGVGCAGGVDVTATRTYQVEETAQDQIGFEINVKGLQGGHSGMDIHKGLGNANKIMNRLLFDGFENFGLRISEIEGGSLRNAIPRESKALVAIDSIHEDAFIMEMAQLSQTIKTELRTMEPQLDISVSKTKVPKLVMDLGVQEGFTKAVYAACNGVYRMSADIPGLVETSNNIAKVLVKDGELKIECLTRSSVESSKWDLANSLRSSFEMTGCDVAFSGDYPGWTPNMDSPILKVLESLYIEMNGEKPHVAACHAGLECGILGQNYPEMDMISFGPNIRGAHSPDERAQISSTQKFWEFVKEILKNIPKK

>61. Line 83325: /product="signal peptidase I"

MTLVQWFIFILIIQVIHGLGTWKLYQKAGRQAWEAFIPVYNAVILMKIINRPWWWTILLFLPIVNLIMFPVIWVETARSFGKSSTTDTWLAILSLGFYNYYLNYAGDNLTYIENRDLHPKTATGDWVSSILFAIVAATIVHTYFIQPYTIPTSSLEKSLLVGDFLFVSKFHYGARIPMTTLAAPMVHDTIPVLGTKSYLYNDNAKEEKTSLINKFQLPYLRLPGFQNVERNDIVVFNWPVDTLYNMYKPANRAYKKPIDKETNYVKRCVGLPGDSLEVRNGYVYINGQKNELPDRAKLQFSYKVETKNGPFDNYELSKRYGITDFPHYNQNYTVCYFPGITNETVEKFKNHPNVAKIEPLMREKGEREGSVFPHDPAYNWNNDFFGPLYIPKAGATIELNTDVLPLYKRLITEYENNELQVKGNQIFINGQQTSTYTFKQDYYWMMGDNRHNSQDARSWGYVPMDHIVGKPVFIWMSYNQHGKGIGEKIRWDRLFTTVNGSGKPTSYFIPFLILMVVGYGFNKFRKRKKEQA

>62. Line 84017: /product="prolyl oligopeptidase family serine peptidase"

MKRVLYAWCLFVSAMVWSQKLAQPEVLPQNIVKNTYHGVTIEDPYQYLENLDDPAVINWMKGNAQYANSVLDNISGKQEMLDKMMELINRTSAAISSLRITDDNTYFYLKRVPGEEIFKMYKRDGYEGKETLFFDPNKYKKDSGKTYTINGIAPNVQGDKVAVTLAANGSENPETLIFTSEGEKFKETIELGRGISWLPSGDAFYYNKFHSADVTDMNRKMFTNVYVHQLGTEQEADTIYFSKKEYGTLGVDGMEIPMIIYDKNAGMNVLVIVSVDKNTRAYFQSPSGNSKVWKSLSQRSDLIVDYEVNDKHLYFMTYLDSPNYQIKKVSLQDLDISKATTVVPESKNEIITGMKLTKDGLYYTTMKNGVEAQVYFLAYNSSTPKQLKLPFTAGDASLSNKGSEFSDIWISISGWTSPDKRYLYHPKDDVFEYQPLSTPAEYPELKDLIAKEVMVASHDGVMVPVSIIYNKNIKLDGSNPGVIYSYGSYGNSTSPFFSPITLAYTLYDGVLIVPHVRGGGELGDSWHKAGQKLNKPNTWKDAIAAAEYVIKEGYTSSDKLAIFGGSAGGILVGRSITERPDLFVAAAPMVGAMNTVRMEETPNGPVNTPEFGTVQDPEEFKGLLEMDSYLNLKPGTDYPATLITAGINDPRVIAWQPAKFAAKLQHDNVGDSPVLFLTDFEGGHGGGVALTKALNNFSNVFSFFYWQSGHPKFKMKEPIKD

>63. Line 85329: /product="S9 family peptidase"

MKQSILLIFVLILVSCKNENQTKETETAQREIPQYTIEQFMDNESVGGGSFSHDNSKLLISSNKSGIYNAYTVSTQGGDYTPITASDSTSYFAQSFFPSDDRMLLSADGNGDEIDHIYLRDTTGTLTDLTPEKEAKSQFLDWTEDKQGFYYLSNKRDPKYFDVYKMGINTFKPSLIYQNNDGMNFSAISGDEQYIALSQSLNTNDSDLYLYNMQTKESTKINDQLSANSAQGFSKDNTTLYYTTDDGQEFSYLMAYDLSTKEKSIVVQKDWDVMGAGFTENGTYMVVYVNEDGKNAVEVTETATKQPLDLPDFGQMSITSVSFSDDEKWMRLYVGGSNTPSDLYTYNIENKALHRITNVLNKDINIDDLVTAQVVRYKSFDGTVIPAIYYLPHQASKDHKVPAMVWVHGGPGGQTRQSYFSLLQYMVNHGYAVLALNNRGSSGYGKTFYKMDDLNHGEKDLQDCVEGKNWLQTQPEIDADKIGIIGGSYGGYMTMAALTYTPEEFNVGVNLFGVTNWMRTLKSIPPYWESFRKSLYLELGDPYSADSVRLKRISPLFHTDKVTKPLMVLQGAQDPRVLQVESDEIVAGVRENGVPVEYVLFEDEGHGFVKKENQIKAYSKVVDFLDEYLKNPTKKMDGELPQADMEAVDDVSSQ

>64. Line 85649: /product="M1 family metallopeptidase"

MKHLLLTTLMTLSVLSGYSQGLLEKKENFTHQDTLRGTITPERAWWDLTYYHLDIEVFPETKTIKGTNTIRYKILERNGVMQIDLQEPMQITKVTQNGQRLYVKHDGNAHFVYLKRYQKVGNTEEIVVHYTGKPREAVRAPWDGGFSWKKDQNGNPFIATSCQGLGASVWWPCKDHMYDEVDSMDISVTVPKGLIDVSNGRLKSKVENPTTTTFNWEVKNPINNYGVNVNIGDYTHFSEVYQGEKGPLDMDYWVLSYNLDKAKEQFKQAPQMMDAFEHWFGPYPFYEDSFKLVEVPYLGMEHQSSVTYGNHYQNGYLGSDLSGTGWGLKFDFIIIHESGHEWFANNITNKDIADMWIHEGFTAYSENLFLDYFYGKKAASEYVIGTRRAVSNDKPIIGFYDVNKEGSGDMYYKGANMLHTLRQLIEDDEKWRQILRGLNKDFYHQTVTTKQVEDYLSEKTGYDLTEFFNQYLRDTRIPVLEYEVEGQQIKFRWTNVVENFDMPVIITYDGKKQWIYPKQEWRTAFIASDDVTIDPNFYIIPKKI

>65. Line 86751: /product="peptidase S41"

MKPLRFIILNLLVLCTLNSCFEDLDDNPATDQDIKDFVYRAMFDWYIYKDEMPQLQTNRAESSDYDSYLNSFSSPENLFESLIYERQTIDRFSAITDDYFALEQQLNGTSYHNGMEYGLFRFSSTDTDVYGYVRYILPSTDAENKGLVRGDIFHSVNGTQLTINNYRTLLGTNSYTIALGSYDDNGTPNDVTDDFISDINQQIQLTKVPYTENPVYDYDVFNIGGEQVGYLMYNGFTGDFNNQLNDVFGYFSSNGVQKLVLDLRYNPGGSVNSARLLGSMITGQFNGDAFSSLNYNDQKQNFNSTYNFTNSFNGTSINSLNLNKVYVIATGSSASASEMVINSLSSYIEVVHIGKNTVGKSQASSIIYDSPDYGRQNVNPTHTYALLPLIAITRNAEGLAVPNSGLVPDVEIGEKVNDLGSIGNQMEPLLAAALQVIENGGRQAALTSKGHEIIGDNHDLEPNSQIMIAD

>66. Line 96271: /product="SOS response-associated peptidase"

MCFHTSTTNKVKKLEAFYSVDLVDESMRPYFDTPKYHMNGFSHPNMLVIPQEKSHVLAPGLWGIVPDNKQPDEIKPYFKEAVRYGGGLNAQSEKVFDHFIYRNSIMEQRCVIPVSGFFEPHDHKKKKYPFHIKSKEDSVLSLAGIYTMVGTYLTFTILTKKASPMFEKIHNLKKRQPVILSQEDAHNWLSVDLNQNDISSLLTETYPENRLEAYPVSKDLFSPKVDSNVSTILEKVEYEELSL

>67. Line 97006: /product="carboxypeptidase regulatory-like

MKVWSFIILGLVCFVSFSQTQLTGIVKDNSGNPLVGAVSTIKNTNSQAIIAYAITDELGGFSFQINLEKEGLEIEIQSLGFKKWKKVLSKKNTHLDIVLQESVEQLKEVFLKSDPIRKRGDTIAYNVSTFKNQSDRTIADVIEKMPGLSILPSGQITYMGEPIEKYYIEGLDLMEGKYKLANESISVEDVARVEILENHQPIKVLDSLVGSQRTSINIKLKNNVSLSGKVEGGIGFSPGLLKTEITPFLLSKKRQTFTSYQYNNIGEDLSSYNSDFSPSQSNLPIFDDSKRNFLKIQQLQNPPFSNERWLNNKDHFGSANHLERLKKGLDLKVNLSYLNGTRKENGFQESIYKNASNNLNYFESIENTFRLHSLNTKITLEKNVKKNYFKNVFSSNNYWDKNHGFIKNNITSISQQALNPFWNLKNQFHILKPMGKQLLEFNSSVGYVNTSQELMVTPGVFIDYFNNGENYEKTHQFLNYHTFYTHNSIGFTKRIGVFTIVPTAGFSFIKEEFKSDIITGVNNNPLTETTNDYGLRSFTTFLTNKFEYKDNIWRITLRTPLFLRNIESPEYNIASKPLTFEPDLFLSKKLTPFWETQFSGQISNSFGEAENLHRGLILENYRSLTSNEPVLSKQIQHRGSLALRYRNSLKGLFGSVSISLTKIKNNLLFDYRVSSNGAITIFSLEQDNNQVQNNMALNLSKYIDDWKTTLTGSSSFFVFKRDQIVNNVFDKYKTIGKQFAFKADIEANKWLTIGLNTQLKTSQLKSKDVSFERVKNWSNSLSAFFYLSKRQFLNADFEHFYNNLQSNQNTVFLNIQYQYSFKKHPIDVKLSWNNILNTKSYINMQNNEYYSLQSSYIIRPSQVFLTLLFTL

>68. Line 97115: /product="M23 family metallopeptidase"

MSKVKYYYDPETLSYRKIERKKRRTFKYISVFLLASALFGFLSVFISSQYIESPKERALKRELSNLQLQYQLINKKMNEAEAVLSSIEDRDNNIYRLYFEANPIPEEQRRAGFGGINRYKDLEGFNNSKLIIETNKRLDILQKQIVVQSKSLDEIAKLAEEKEKLLAAIPAIQPVRNEDLRRMASGYGVRSDPFTKARKMHWGMDFSAPRGTPIYATGDGVVTRADNRATGYGNHIEIDHGYGYVTIYGHLYKYNVKKNQKVKRGDLIGFVGSTGRSEAPHLHYEVHKDGERINPINFYYGSLTAEEFDELLQHASMENQSLD

>69. Line 102778:/product="M1 family metallopeptidase"

MIWGQNRMDIKADFDTENKQIKIEQTITYINTSNDVLTEIYLNDWIQSFSSKTTPLAKRFAEEYKTVFHFAKSEDRGYTAITAMTQNGQELSFNRLKDQPDVIKVIPITPIKPNESYTIELKYVVQVPNDKFTRYGVSDLGEYHLRYWYITPAIYNGKWQYYSNKDLDDMFIPKSDISMEVRFPKDLYLASELNVLSTKNDGNKQIITLEGKDRINTKLFLDRLKRFSQVETEYFTLIYNIDNENLNLVDQLINIDKIAGFLDQNLGSYPHENLLLTHIDYKKNPIYGLNLLPDFIRPFPDQFQYELKILKTALNNYLENILLINPREDQWLIDGLQTYFLIKYVDENYPEMKIFGTLSNVWGIRTFHAAELDFNDQYNFLYMHMARTNLDQPLLMQKDSLLKFNMNIANKYKAGVGLRYLDDYVNANILENTIKEFIANQSLKETNTGDFEQLIKSKTSKNIHWFFDEYLKTSKKIDFKIKEVQEFPDSIQVTIKNKRDNHMPVSLFGLRNDSVVYKTWVDDINKSKTVTVPKDHIDKLVLNYDKSIPEFNLRDNWKSMKGFLFNNKPIQFRLFKDIEDPYYNQIFFMPQIKYNYYDGFAPGLKLYNKTVLTKAFNYNLRPSYALKSQQLVGSGNVSYIQRPENRDLYYIRYGFSGAYSNYAPNLSYTSLQPYVQFRFRDHKDLRNNRKNYLTFRYVSINREEDPLGIYNVEEEPSYGVFNAQFGHSNPNLKNYKFWNTDLQLAKNFGKASFNYEFRTLTEGNRQYNLRFFGGVFLYNKTYENSNFFSFALDRPTDYLFDYNYYGRSENTGIFSQQLIIAEGGFKSKLNPAYANQWITTLNGSATIWKYIHAYGDIGLVKNHNFNPEFVYDSGIRFSLVEDYFELYFPVYSNLGWEVAQPNYEEKIRFIVTLDIKTLFGLFTRRWY

>70. Line 103311:/product="M1 family metallopeptidase"

MKPYLNLFFAIFSLFVYSQQSYNVDFKSVLVDLSFNTLEKLVMGNLTYEFEILKPVDSIYLDAVGMIFKDVKLNKSTAKWDNDGDKFWLLERLVPNKKYQLSFTYQVSPKKALYFVGWDNEAPNQIWTQGQGKYTSHWLPSLDDTNDKIEFDLQVTFDKAYEVVANGNLVNKKEKNGLITWTYDMERPMSSYLVALVIGDYDKKEEISKSGKPLEMYYYPKDSMRVEPTYRFSKTMFDFLESEIGFAYPWQNYKQIPVHDFLYAGMENTSATIFADSFVIDSISFVDKNYVNVNAHELAHQWFGDYVTAKNGDHHWLQEGFATYYALLAEREVFGEHYYFQRLYEYYQELMQQERAGQGTSLLNPKSSSLTFYKHGAWVLHALKDKVGDEAFEKAVRNYLNKHAYGLVETNDFISEVEQASGLNLHGFQLLWIENVKFPQEQAYELLMKSAYIQEYEMADCEMNSGKCAYYLNSGVSDWTKSKVIAQVQDKVTVDLFNSGWEVRQAIAESVSKIPQNLKESYESLLNDPSYKTIEAALYHLWINFPEDRATYFKQTKDLVGFPDKNIRLLWLALALSSPNFEPENNETYYYELLDYTSPRFGFEVRLGAFQYLDALKGCNDICQDNLKQATKHHNWRMSKFAKEMLERLKPVQN

>71. Line 109756:/product="endopeptidase La"

MKKTSILNLDSLSLQDFDENSELIPLMTPEDEEEINKEELPETLPILSLRNTVLFPGVVIPITAGRDKSIKLINDANKGGKVIGVVAQKDEAIENPTAKDLHQTGTVARILKVLKMPDGNTTVILQGKKRFQINQVITEEPYITATIEGVSETKPEPANKEFEAIIESIKDLSLMIIKESPNIPSEASFAIKNIQSNSFLVNFVSSNMNLSVQEKQSLLESSDLKDRALETLKFMNLEYQKLELKNDIQLKVQNDMNQQQREYFLHQQMKTIQEELGGVSYDEELEEMKERSKSKKWDEKVAKHFEKEIAKMHRMNPQVAEYSIQRNYLDLFLDLPWNEFSKDNFDLKRAKRILDRDHYGLDDVKKRIIEYLAVLKLRNDMKSPILCLYGPPGVGKTSLGKSIAEALGREYIRMSLGGLRDEAEIRGHRKTYIGAMPGRIIQNLKKAGTSNPVFVLDEIDKLSNSHQGDPSSAMLEVLDPEQNSDFYDNFLEMGFDLSKVMFIATSNSLATIQPALRDRMEIINVTGYTIEEKVEIAKRHLLPKQLKEHGLTAKDLKIGKTQLEKIVEGYTRESGVRGLEKQIAKMVRYAAKNIAMEEEYHVKVTNDDVIEVLGAPRLERDKYENNNVAGVVTGLAWTQVGGDILFIESILSKGKGQLNITGNLGKVMKESATIAMEYIKAHADEFNLNPEIFDKYNVHIHVPEGATPKDGPSAGITMLTSLVSLFTQRKVKKNLAMTGEITLRGKVLPVGGIKEKILAAKRARIKEIVLCEENRRDINEIKEEYLKGLTFHYVSDMSDVLKIALTNQKVNNAKEL

>72. Line 109888:/product="M48 family metallopeptidase"

MNIKNKIVAFGGLLLFLSCATNPFTGKQTMAFVANDQIFPASFQQYNQVLSESKVVKGTSEAEMIKRVGERIATASERWLNALGYQGYLKDYRWEYNLIQSDVVNAWCMPGGKIAFYTGILPIAENETAIAAIMGHEVAHALVNHGQQRMSAGMLQQAGAIGLNVFLQDNENLELFNQAYGIGSTVGGMLPFSRAHEEEADKIGLYIMAIAGYNPDEASRLWERMGASGGQSPPEILSTHPSTDSRIANLKALAPKAKAEAQKFGVTSYRPITKF

>73. Line 110250:/product="M23 family metallopeptidase"

MKYTLALFLLCSPLLKAQNIYPQDYFQSPLDITLVMAGTFAELRSNHFHSGIDIKTQQVEGLKIYAAADGYISRIKIAHFGYGKAIYITHPNGYTTVYAHLQRLSPTLEAYIKKLQYEQESYEVEVFPNPDELIVSKGEVVAYSGNTGSSGGPHLHFEIRDNAERPINPMLFGLDIKDTTPPFIKNVYAYPIGPDSHINKSNEKQKLRLIRQPNNDYVVENVEAYGKIGFAIEANDRQDLAYNNNGLYNIQTFLNGNQKIEIDFHKFSFDESSHINRFIDYEQYINEKHRLQKLYRDTNNPLSIYKNLDDEGYLQIQDSTSSIYKVKVSDYKNNNTWLTINIKGKKYDTIKKEEPFKSNYFIYANKTNELVHDKVAVEFPSNTFYEDFYMDFAQTNDTIKLHDESIAAQKYFTITYDLSHYKPEDLGQLYIGRLVGYYKRPYYSSTKKEGHILSTRTKTLGIYALAKDTVDPTIKPINFSDGKWVSNFRYLKLKIEDKESGISKYRATVNGKFILMEYEYKDDMLTFDFNDNVITETENNLKVIVTDNVGNSSTFEATFYRK

>74. Line 110806:/product="M42 family metallopeptidase"

MAKKQVLNKKSLDFLEKYLNNAAPTGYEWDGQKLWMEYVKPYVDTFITDTYGTAVGVINPDAKYKVVIEGHADEISWYVNYITDNGLIYVIRNGGSDHQIAPSKIVNIHTKKGIVKGVFGWPAIHTRDKSKEQTPSLENIFIDVGAKDKAEVEKMGVHVGCVITYPDEFHILNGDKFVCRALDNRMGGFMIAEVARLLHENKKKLPFGLYITNSVQEEIGLRGAEMITQTIKPNVAIVTDVTHDTTTPMIEKKKEGHLELGLGPVVAYAPAVQQKLRDLITDTAEENKIPFQRSALSRATGTDTDAFAYSNGGVASALISLPLRYMHTTVEMVHKEDVENVIRLIYESLLKIKDGESFSYFD

>75. Line 114893:/product="ATP-dependent Clp endopeptidase proteolytic

MDYAKEFEKFAIKDQGISSTYYNKIMSSMYPVGLTPNIIEERQMNIAIFDVFSRLMMDRIIFLGTAINDQVANIIQAQLLFLESTDSSKDIQIYINSPGGGVYAGLGIYDTMQFIKPDVATICTGMAASMGAVLLCAGKEGKRSGLPHSRVMIHQPLGGAQGQASDIEITAREILKLKEELYQIISKHSGQPYDKVYDDSDRDYWMKAEQAKEYGMIDEVLMRS

>76. Line 115752:/product="signal peptidase II"

MKFTRNVGIILLVIFNIALDQISKFIVRAKVDPYSETPIIGDYFTLHNVENKGAFLGMGSELNDTLRLILLLILPLAVLGYVMYYIITNKNLDKASVIGFSCIVGGGIANVYDRVMYGSVTDFFHIDLGGVFRTGIFNIADMSVMFGLGLLLYSNFKNKKASN

>77. Line 115967:/product="carboxypeptidase-like regulatory

MKYNLLAFLCLLGITFGFAQSKVSGYVMDEKDEPIAYANVFFKSSSEGTITDENGKFYLQSDNTWETLVVSFVGYETIELPLEKKVNYNLKFTLIEEAAALNEVVIYTGKQSKKEEENPAIAILKKIWARKRFNGLKQFKQYEYDKYEKIEFDLNTIDSALMKSKLFKGMEFVFEQTDTSSVTGKTYLPMFINEAVSKVYGDNTTNKVKEQLKGNKNSGFDNNQIVIDFIDDLYSDYNIYDNYLKFFDKSFVSPISRTGINTYNYVLIDSAFVDNKWCYNIVYYPRRKNELTFKGDFWVNDTTFAIKNINMHASKSANINWVKDIYIEQDFEVLNDSVFLIKRDYFMSDFSFQKKEKARGVYGKRTTLYDNYVFNFKRPESFYKEEVYNPNDQAFERDSTFWQKHRLEELNKDEKGIYTMLDTLRTVKKFKRLYSIGEILASGYIELDEINMDYGPIFSTFGFNEVEGMRIRTGGRTYFTPHDPWRIEGFAAYGFKDQKFKYGISLKAMIEKKNRLIISGGYRKDVEQIGVNLTSSQDVLGRSLASSSLVTTSPNDKLTKIELGGLAVDFEPIKNLEFRITGNYRVLESASPTFSLAYRDPEAPNGVNDVVKQYESTVSLSYYPNRRMTGYGVERYVVNDYFARLFVQVARGDKGLLNSDFDYTKLQFSYMQPWRIGGLGVLNTTLEAGKTFGDVPLGLLSVIPGNQSYFSIYNTFSQLDFYEFVTDTYASLHAEHNFNGRIFSRIPFLRKLNLREIVGFRAAWGDISDANIYLNEPNGIPLIAPTDKIYYEYSFGVGNIFRVFRIDFNFRGNYKDAPNARDFGVTGTFGFNF

>78. Line 116043:/product="M1 family metallopeptidase"

MKRIFLVLNLLFLPLLINAQKQPWQGKFEPIDNLITPPSTYRTASGAPGRDYWQQRADYNIKASLDEKTKVLSGEETITYYNNSPDELTYVWMQLEQNVNKKEGESFGDAFGGVRDSISSLNMQYLTRAIEFPAGHTIKYVKNASGKDLKTLVNQTMMKVFLDSPLKSGETTQFQISWSYPITDRSMFMLSREGYEYFPKDDNAVFLIAHWFPRMCVYNDTEGWQNKQFRRLGEFALEFGNYNVEITVPEDHVVASTGSLQNEEQVLSKQQRKRLDEAKKSFGAPVLIITPEEALKNEQSKSSKMKTWKFKAENVRDFAWATSRKFIWDAQAHQFSDHVTMAMSFYPNEGLPVWEEESTKAVVAALEVYSEATFEYPYPVAISVNTSNIGMEFPMISFNGGRPKNGKISDMAKAGMIGTIIHEVGHNWFPMIVSSDERQWMWMDEGLNTFLHQRTVAEKYPQFSHTTPKSIVPFMNGNQDIMRPIMTSSDNELLTQFGANFYQKPTVGLQILRNSVIGKELFDQAFKEYANRWKYKHPNPADLFRTLEDATATDLDWFWRGWFFTTDKTDMELSQVKWFRVEEKDQNIEDQQKKTKIQVEGQDTKTVATDFSGGPEMITMTETPDNNYGGFLSRLDETQIRNNLSGKNIYEVTIKNVGGLVMPVTIEWQYTDGSSETDRLPAEVWRYNEYEITKTFVKDKEVKQVILDPNFEFADVETANNSFPKTEEASQFDKFKEKKN

>79. Line 116419:/product="P1 family peptidase"

MKRISIFIITLLLCFYSHAQKPRARDLGVPFVGVPGLNNAITDVKGVEVGYSTIISGEGENVIGKGPIRTGVTAIFPRGKAKKFSPVYANWYSLNGNGEMTGTTWVTESGFLETPIMITNTNSVGEVRQAVLKWFVDTDWYRGENWWYTYPVVAETYDGFLNDIYGFHVKENHVLEAIKNASSGKIAEGNVGGGTGMMCLGFKGGTGTSSRVFEINDTQYTVGALVQSNFGAKRNLSIAGVPVGIELKDTLNYEYKAPPKSSRQEGDGSIIVIVATDVPLLPHQLKRIAQRIPLGVGIVGGRGSNGSGDIFLAFSTANEKAFNRGEMTTVQTMSNDLLMPVFEATVQVVEEAIINAMVAAETMEGINGNKAHALPHDLLIETLKKYNRIED
